# Behavioural and cognitive changes in young adults towards food and nutrition after exposure to digital food communication: a mixed-methods systematic review

**DOI:** 10.1186/s12966-025-01817-5

**Published:** 2025-10-20

**Authors:** Shaniek Parks, Asha Kaur, Jessica Renzella, Reem Malouf, Ornella Moreno-Mattar, Charlotte Albury, Mike Rayner, Peter Scarborough

**Affiliations:** 1 Nuffield Department of Population Health, University of Oxford, Oxford, United Kingdom; 2 Nuffield Department of Primary Care Health Sciences, University of Oxford, Oxford, United Kingdom; 3 Johns Hopkins University - Pompeu Fabra University Public Policy Center, Universitat Pompeu Fabra (UPF) - UPF Barcelona School of Management (UPF-BSM), Barcelona, Spain

**Keywords:** Young adults, Food and nutrition, Digital media, Health behaviour, Media exposure

## Abstract

**Background:**

Young adults (18–25) face significant risk for weight gain and transitioning to a higher body mass index category when compared to other adult groups. As active internet users, they encounter food-related content across digital platforms, yet little is known about their behavioural and cognitive responses to this compound exposure.

**Methods:**

This pre-registered mixed-methods systematic review features primary studies about participants aged 18 to 25 exposed to digital food communication and assessed for behavioural or cognitive responses towards food and nutrition. We evaluate consumption and food purchase as behavioural responses; intentions to consume and/or purchase, and attitudes towards food and nutrition as cognitive responses.

We searched PubMed, PsycINFO, Global Index Medicus, and Business Source Ultimate for studies published from database inception to August 1, 2024. Quality appraisals were conducted using a modified version of the Newcastle-Ottawa Quality Assessment Form, the ROB1 Tool for randomised trials, the JBI Quasi-Experiment Checklist for quasi-experiments and the Critical Appraisal Skills Programme (CASP) checklist for qualitative studies.

We used a three-pronged method for analysis. Meta-analyses combined findings from randomised trials for behavioural and cognitive responses, while observational studies were summarised narratively. The thematic synthesis approach informed our qualitative synthesis of young adults’ narratives of their responses after exposure to digital food communication. Finally, a cross-study matrix synthesised analytical qualitative themes and quantitative findings.

**Results:**

Of the 6132 studies identified, 45 are included in the systematic review, representing 8,914 young adults in 16 countries. Meta-analyses on behavioural and cognitive responses demonstrate statistical significance with effect sizes of 0.34 (95% CI: 0.18-0.50) and 0.19 (95% CI: 0.10-0.28), respectively. Observational studies confirmed the findings of the meta-analyses. Difficulty deciphering what represents good nutrition, critical distinctions when engaging with content viewed as helpful versus misleading and balancing intentions versus actual behaviours were barriers to the effectiveness of digital food communication. Using a cross-study synthesis matrix, we developed ten recommendations to improve digital dietary interventions and their assessed implementation by experimental studies in the review.

**Conclusions:**

Our results illustrate the need to approach digital food communication as a digital determinant of dietary health for young adults, shaping behaviours and cognition.

**Supplementary Information:**

The online version contains supplementary material available at 10.1186/s12966-025-01817-5.

## Background

Young adults aged 18 to 25 live in a developmental phase called ‘emerging adulthood’ marked by the transition from adolescence to adulthood [1–4]. Key changes include increased autonomy in decision-making, new financial responsibilities, and shifts in support structures and interpersonal relationships [5]. From a health behaviours perspective, this life stage is characterised by lower diet quality compared to other adult age groups [6, 7] and an increased risk of weight gain [5, 8]. A longitudinal study involving more than two million people identified young adults aged 18 to 24 as being at the highest risk for weight gain and, if overweight or obese, were at the greatest risk of transitioning into a higher body mass index category when compared to other adult groups [8].

Young adults are active internet users, with many relying on health information found online [9–11]. Bartelmeß and Godemann [12] introduced the concept of digital food communication as the practice of sharing and interacting with food-related content through social media, which creates new opportunities for people to engage with food-related content online. Several researchers have sought to understand the influence of food-related content on digital platforms on young people’s health behaviours [13–16]. These qualitative and quantitative studies have focused on food-related content across platforms, including social media, websites, mobile applications, virtual reality, wearable devices, video games, SMS messages, emails and online communities. However, there is no synthesis of evidence evaluating the cumulative impact of exposure on young adults.

To understand the cumulative impact of digital food communication on young adults, an exploration of cognitive and behavioural responses can provide complementary and instructive information. Cognitive responses reflect the ‘why’ behind decision-making about food, providing self-reported data on how people evaluate food, which may be through their intentions and attitudes [17]. In contrast, behavioural responses allow us to observe the actions taken, whether deliberate or automatic [17]. The need for synthesis is underscored by the current discourse surrounding the digital determinants of health, spanning information, social media, digital literacy, and the digital ecosystem [18–21].

This mixed-methods systematic review aims to evaluate and synthesise how exposure to digital food communication influences young people’s behavioural and cognitive responses to food and nutrition. Our research questions are:

- What is the impact of digital food communication on young people’s behaviours towards food and nutrition?
- What is the impact of digital food communication on young people’s cognitive responses to food and nutrition?

In this review, we expand the concept of digital food communication to cover all food-related communication in the digital environment. We define digital food communication as a convergence of electronic communication about food occurring on social media, interactive media, blogs, websites and the internet by leveraging digital marketing [22], influencer marketing or engagement, online food communities [23] and activities by “ordinary people as participants in online food culture” [24]. Our definitions for behavioural and cognitive responses are derived from Kaneko et al.’s [17] definitions for behavioural and cognitive measures, which consider lower, intermediate, and higher emotional processing levels. We define behavioural response as a response reflecting the implicit and/or explicit response of the body. We define cognitive responses as explicit opinions, choices and decisions involving cognitive processing, which may be unknowingly influenced by an implicit response. Here, we evaluate behavioural responses as food consumption and food purchase, and evaluate cognitive responses as the intention to consume, the intention to purchase and attitudes towards food and nutrition. Additionally, we selected a mixed-methods approach for the systematic review to enable inclusion of a broad range of relevant studies on the topic [25], to gain a comprehensive overview of the current state of digital food communication and its influence, something a single-method review would not achieve due to its limited perspective [26], and to utilise the diverse dataset to develop actionable recommendations for researchers and policymakers [27].

## Methods

The mixed-methods review was pre-registered with the International Prospective Register of Systematic Reviews (PROSPERO), CRD42023412468 and followed the Preferred Reporting Items for Systematic Reviews and Meta-Analyses (PRISMA) 2020 checklist and the Enhancing transparency in reporting the synthesis of qualitative research (ENTREQ) guidelines.

### Eligibility criteria

The review included primary, peer-reviewed articles about participants aged 18 to 25 exposed to digital food communication and assessed for behavioural or cognitive responses to food and nutrition (Table 1). We used MeSH terms to define the core concepts of food, population, and digital food communication. We did not apply MeSH terms or keywords relating to behavioural and cognitive responses. Instead, we used predefined conceptual definitions to guide the screening process. Specifically, we adopted Kaneko et al.’s [17] definitions identifying behavioural responses as food consumption and food purchase, and cognitive responses as intention to consume, intention to purchase, and attitudes towards food and nutrition. This approach was developed in consultation with a health information specialist librarian to ensure a comprehensive and inclusive review strategy.

**Table 1:** Inclusion and exclusion criteria

Inclusion Criteria	Exclusion Criteria
1. Published in English or Spanish	1. Not published in English or Spanish
2. Peer-reviewed academic literature	2. Studies not published in academic peer-reviewed literature (e.g., conference papers and dissertations)
3. Primary research only	3. Existing reviews of data (e.g., systematic reviews, literature reviews and scoping reviews)
4. Studies any population where the median or average age falls between 18 and 25	4. Studies any population where the median or average age falls outside of 18 and 25
5. Studies that contain digital food communication as an exposure variable, such as the use of social media (social networking websites, online communities, online influencing, digital marketing), engagement on social media (liking, sharing, following accounts, saving posts, commenting and creating content), image or video-related activities posted online, online communities, digital interventions, blogs and websites.	5. Does not include digital communication (e.g., TV advertising, radio, print)
6. Studies focussed on food, nutrition and/or non-alcoholic beverages. This includes studies on nutrition that contain dietary supplements, vitamins and mineral intake	6. Does not contain food or non-alcoholic beverages (e.g., studies on alcohol, cannabis/marijuana, drug use and tobacco-related products)
7. Studies that contain a behavioural or cognitive outcome(s)	7. Does not contain a behavioural or cognitive outcome(s)

Due to the language proficiencies of the systematic review team, articles written in English and Spanish were included. Our criteria for digital media aligned with recent literature that distinguishes digital media as a distinct driver of food preferences and consumption behaviours among children and adolescents, warranting focused investigation separate from traditional media channels, such as television, radio, and print [28]. Studies comparing one form of digital communication to another were excluded, as were articles that used secondary data. Cross-sectional studies had to evaluate the association between digital food communication and cognitive or behavioural responses. Research designs were not restricted.

The S.P.I.D.E.R. (Sample, Phenomenon of Interest, Design, Evaluation, Research type) tool was used to define study eligibility criteria due to its suitability for mixed methods evidence synthesis [29]. The S.P.I.D.E.R tool, in the supplementary materials (Table 1), presents review definitions.

### Information sources & search strategy

The search strategy was developed with the support of a health librarian, piloted on PubMed and refined to improve the relevance of search output. We conducted searches on PubMed, PsycINFO, Global Index Medicus, and Business Source Ultimate for articles from the inception of the databases to April 18, 2023, and updated the search on August 1, 2024. The finalised Boolean search is located in the supplementary materials (Table 3). Database outputs were supplemented by searching the reference section of included articles to find previously published articles that would meet the criteria for inclusion in the systematic review.

### Selection process and data collection process

Independent researchers evaluated the eligibility of each study at abstract-title screening, full screening, and data extraction using Covidence. Each mixed-methods study was considered for qualitative and quantitative research analysis.

Three people conducted abstract-title screening: OM-M, DNG-T and SP. OM-M and DNG-T are native Spanish speakers who are fluent in English, and SP is a native English speaker. We resolved disagreements during a joint meeting and involved a fourth reviewer [SW] when necessary. OM-M and SP conducted full screening and data extraction of studies. We met to resolve disagreements. SP extracted data using Microsoft Excel, Microsoft Word, and NVivo.

Extracted data for quantitative studies were the study design, country of research, population and mean/median/average age, sample size, digital media, exposure, intervention, control, outcome and results. For studies included in the meta-analysis, the direction of effect was also captured. Extracted data for qualitative studies were the study design, country of research, population and mean age, sample, digital media used and details in the results section of articles.

### Synthesis of methods

**Fig. 1:**
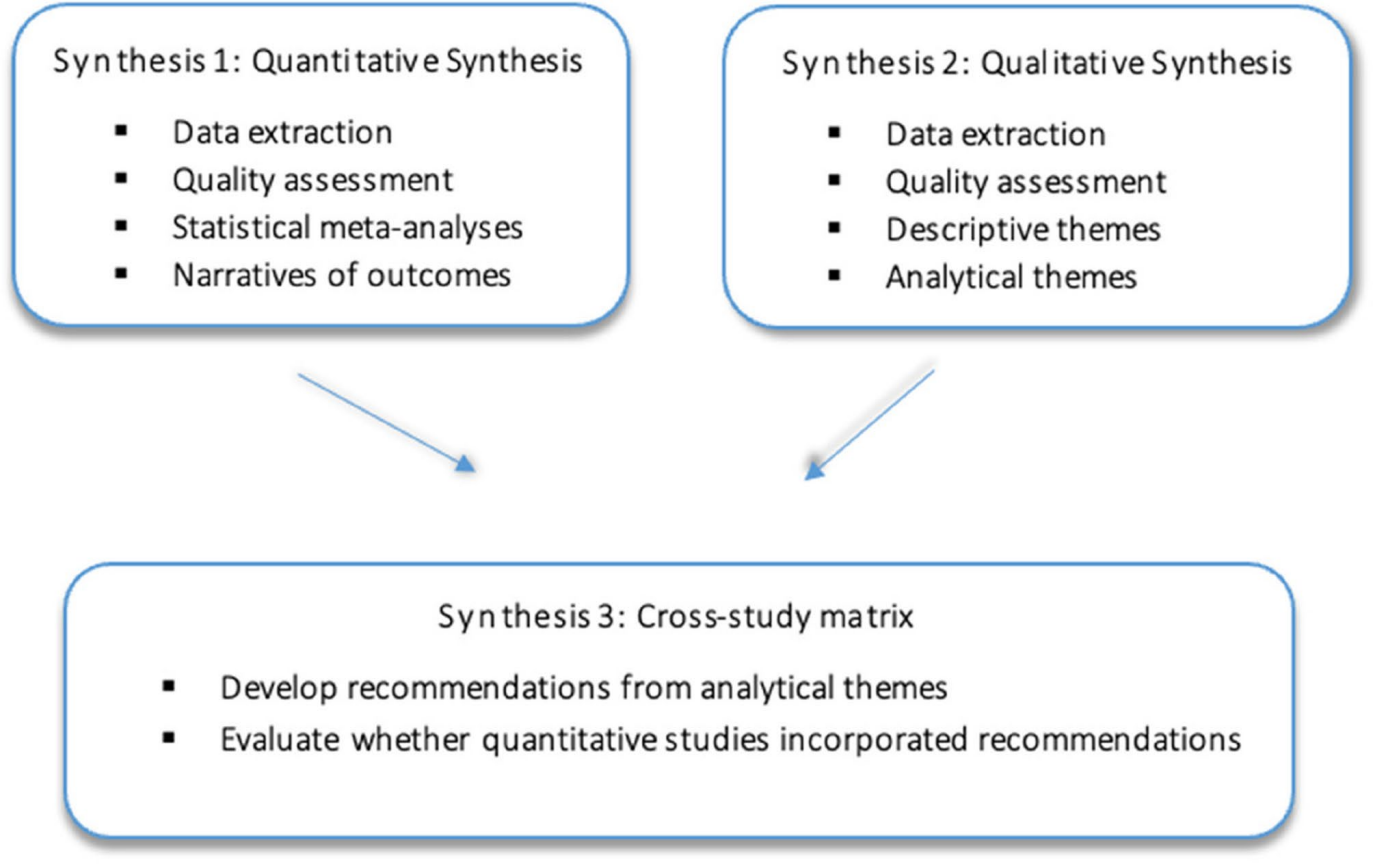
Mixed-methods synthesis approach developed based on Harden [27]

Mixed-methods synthesis, shown in Fig. 1, is a three-stage process in keeping with the approach of Thomas et al. [30], Thomas & Harden [31] and Harden et al. [27]. Quantitative and qualitative findings were analysed separately during the same phase of the research process before using a cross-study matrix to combine both quantitative and qualitative findings.

#### Synthesis 1 – Quantitative synthesis

Data from quantitative study designs were analysed by the behavioural and cognitive outcomes. Individual studies could have multiple cognitive and/or behavioural outcomes. For studies with multiple intervention arms, the most comprehensive arm of digital food communication was selected for analysis. Random effects meta-analyses combined continuous variables in randomised trials for behavioural and cognitive outcomes. The standardised mean difference was used to measure the difference between intervention and control groups post-intervention. Where possible, standardised mean differences (Hedge’s G) were estimated using the Cochrane Revman calculator. Study authors were contacted to request necessary statistical information missing from the publications. Adjustments were made for multiple outcomes from single studies using the multilevel method in Stata 18. Randomised trials that did not present findings as continuous variables and quantitative observational studies are summarised narratively and in tables. These tables reported whether each study confirmed its hypothesis and whether the test achieved statistical significance using a threshold of *p* < 0.05.

#### Synthesis 2 – Qualitative synthesis

Using the results sections of all qualitative papers, we took the thematic synthesis approach [31] to code, synthesise and interpret findings. The approach has three stages: coding the results from original studies, the development of ‘descriptive themes,’ and the generation of ‘analytical themes’. We considered the review’s research questions while coding and conducted ‘balancing of the results’ by considering the rigour and flexibility of the information published, and our interpretation of what each study’s author presented. Similar codes were grouped to form subthemes, and subthemes were grouped to form themes. Descriptive themes were discussed with members of the systematic review team to draw in additional perspectives, before developing analytical themes.

#### Synthesis 3 – Cross-Study matrix

Analytical themes from the qualitative synthesis were used to generate recommendations for future digital food communication interventions and campaigns targeting young adults. Armed with these recommendations, we evaluated whether the included quantitative studies incorporated these recommendations. Evaluation of whether the included quantitative studies incorporated the recommendations was conducted by the main author. As with all data collection throughout this review, evaluations utilised information published within individual studies and noted by the evaluating researcher. The method of analysis was discussed with members of the systematic review team, and the results of this analysis were shared and feedback was incorporated.

### Study risk of bias assessment & reporting bias assessment

This review used several appraisal and risk of bias assessment tools. Qualitative studies were appraised using the Critical Appraisal Skills Programme checklist [32]. Quantitative studies were assessed using a modified version of the Newcastle-Ottawa Quality Assessment Form for cross-sectional studies, the ROB1 Tool for randomised trials [33], and the JBI Quasi-Experiment Checklist for quasi-experiments [34]. S.P. and OM-M conducted risk assessments and appraisals.

Funnel plots assessed reporting bias in meta-analyses involving at least ten studies. We employed Egger’s linear regression test to examine potential publication bias and measured heterogeneity using the I² statistic.

### Certainty assessment

In keeping with recommendations from the Cochrane Qualitative and Implementation Methods Group, we conducted a confidence assessment of individual qualitative descriptive findings using the Grading of Recommendations, Assessment, Development and Evaluation – Confidence in Evidence from Reviews of Qualitative Research (GRADE-CERQUal) approach [35] with the Interactive Summary of Qualitative Findings tool (iSoQ). Each descriptive finding was assessed as having no/very minor, minor, moderate, or serious concerns using four of the CERQUal components, namely, methodological limitations of the studies that have contributed to the individual review finding, coherence of finding, adequacy of data and relevance of the evidence synthesis. Descriptive findings received an overall assessment of the confidence level with an explanation. The confidence level in each finding was also reported as high, moderate, low, or very low.

## Results

**Fig. 2:**
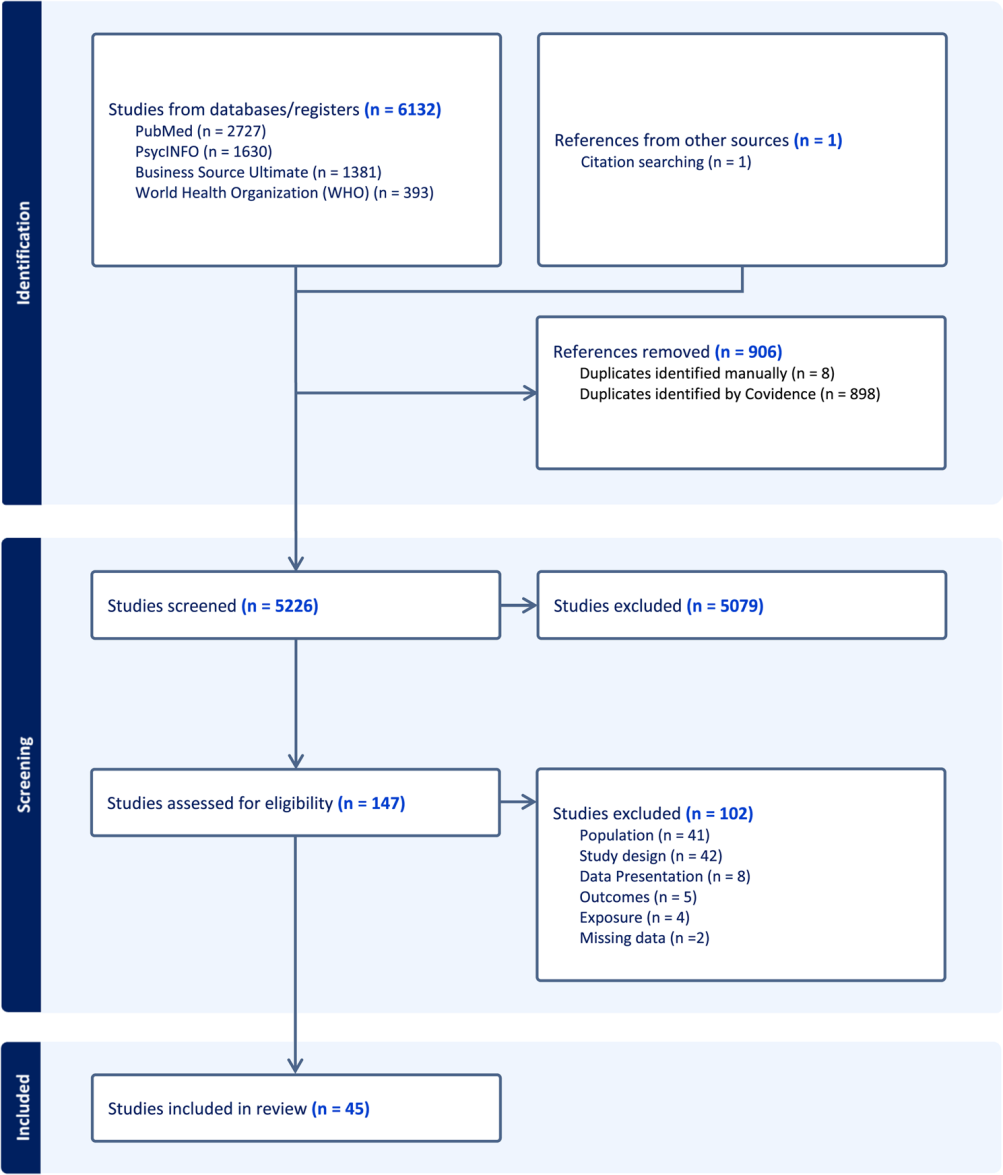
PRISMA chart showing a summary of the search and review process used in the systematic review

The pre-registered protocol is detailed in [36]. Database searches identified 6,132 articles, with 906 duplicates. After the title and abstract screening of 5,226 studies and full-text screening of 147 publications, 45 articles fulfilled the inclusion criteria for the systematic review. The reasons for article exclusions at full screening are located in the supplementary materials. Figure 2 shows the PRISMA Flowchart.

### Study characteristics

The review includes 45 studies published from 2011 to 2024. Of this number, 31 studies provided only quantitative data, 12 provided only qualitative data and two mixed-methods studies provided both qualitative and quantitative data. Detailed characteristics of each qualitative and quantitative article are in the supplementary materials (Tables 3 and 4).

The quantitative study designs used were randomised trials [*n* = 24], non-randomised trials [*n* = 3], quasi-experiments [*n* = 3] and cross-sectional studies [*n* = 3]. The qualitative study designs used were focus groups [*n* = 4], interviews [*n* = 5], digital ethnography [*n* = 4] and an end-user co-design workshop [*n* = 1].

The tools used for digital food communication in the studies reviewed were social media [*n* = 24], websites [*n* = 12], mobile apps [*n* = 10], virtual reality [*n* = 3], wearable devices [*n* = 3], video games [*n* = 3], SMS messages [*n* = 2], and emails [*n* = 1]. Several of the included studies [*n* = 9] utilised two or more of the aforementioned tools for digital food communication. Of the 45 included studies, [*n* = 2] featured only male participants, [*n* = 6] featured only female participants, [*n* = 33] had male and female participants, and [*n* = 4] did not specify genders. Most studies [*n* = 25] utilised student participants from universities and colleges, while others [*n* = 20] selected participants from the general population. The number of participants in individual studies ranged from 1 to 2269.

Sixteen countries are represented in this review. They are Australia [*n* = 11], U.S.A [*n* = 9], China [*n* = 4], Netherlands [*n* = 4], United Kingdom [*n* = 4], Canada [*n* = 2], Brazil [*n* = 1], Denmark [*n* = 1], Finland [*n* = 1], Germany [*n* = 1], India [*n* = 1], New Zealand [*n* = 1], Singapore [*n* = 1], South Africa [*n* = 1], South Korea [*n* = 1] and Taiwan [*n* = 1]. One study conducted with users on Twitch (a live-streaming video platform) did not specify the location of eligible participants.

### Risk of bias in studies

Qualitative studies were appraised using the Critical Appraisal Skills Programme (CASP) Checklist, which showed that five studies fulfilled all the CASP criteria, five left areas of uncertainty, and four did not provide all the information. Randomised trials were evaluated using the Risk of Bias 1 (ROB1) tool, which showed that five had a high risk of bias, 14 caused some concerns, and five had a low risk of bias. The modified Newcastle Ottawa Scale (NOS) was used to evaluate cross-sectional studies, of which four were high-quality and one was fair quality. Quasi-experiments were assessed using the Joanna Briggs Institute (JBI) Quasi-Experiment Checklist, with all six studies appraised for inclusion. The JBI Checklist allows researchers to include, exclude or seek more information after appraising articles. All risk of bias/quality assessments are located in the supplementary materials (Tables 12, 13, 14 and 15).

### Results of individual studies & synthesis

Synthesis occurred in three stages. Synthesis 1 is the quantitative synthesis; Synthesis 2 is the qualitative synthesis and Synthesis 3 is the cross-study matrix that combined quantitative and qualitative findings.

## Synthesis 1: Quantitative synthesis

### Behavioural outcomes

Twenty-six studies reported on behavioural responses, comprising data from 6,880 young adults. Several studies aimed to increase the consumption of nutrient-rich items such as fruits, vegetables, calcium, and vitamin D, while others targeted a reduction in sugar-sweetened beverages and energy-dense, nutrient-poor foods. For example, Ashton [37] measured increases in fruit and vegetable intake, while Kerr [38] examined reductions in sugary drink consumption. In our meta-analysis, we assigned a direction of effect of 1 to studies targeting increased consumption of healthy foods, and − 1 to those assessing decreased consumption of unhealthy foods. Details on how behavioural responses were assessed in each study are included in the supplementary materials.

#### Meta-analysis

**Fig. 3:**
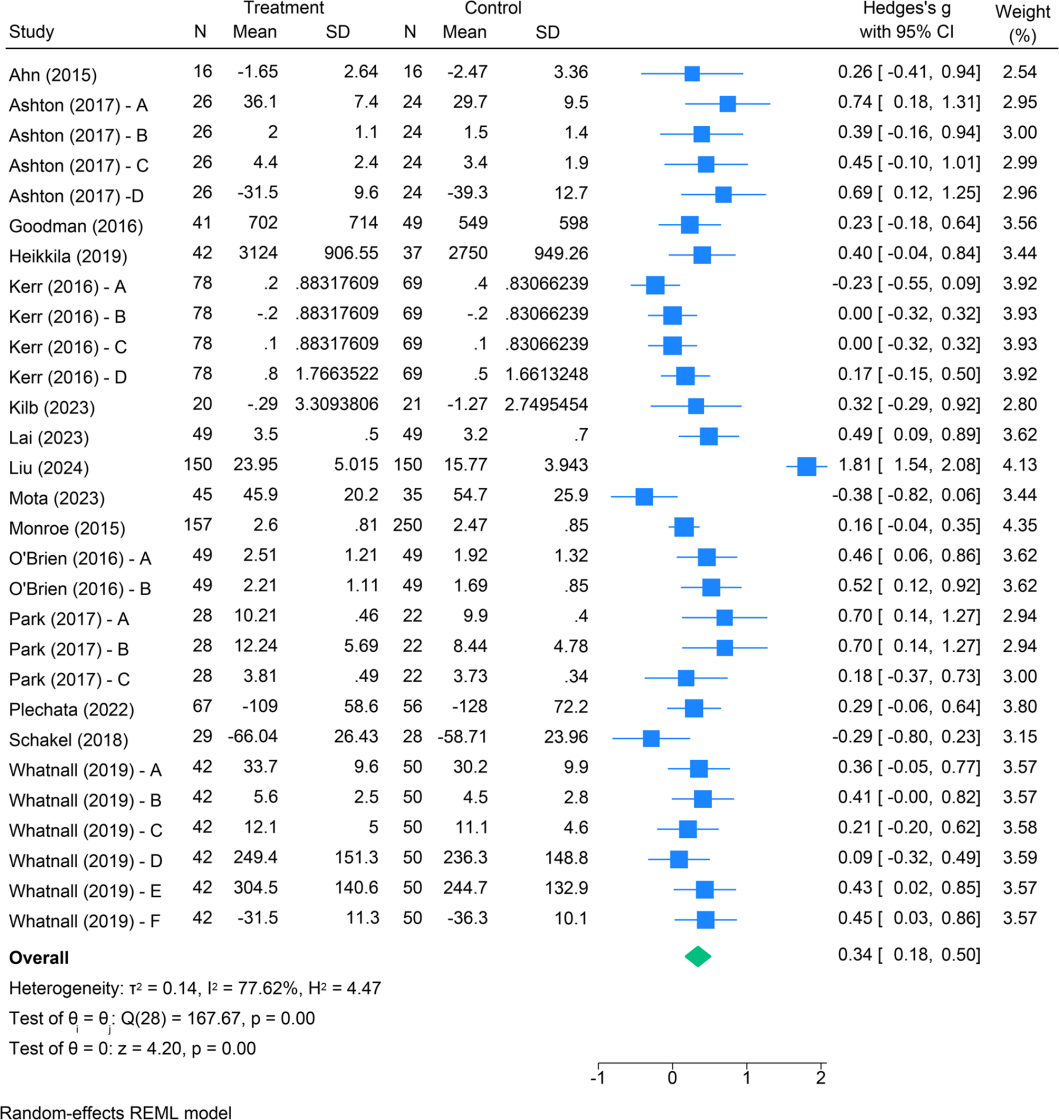
Impact of digital food communication on behavioural responses to food and nutrition

The random-effects meta-analysis of behavioural responses included 1,744 participants from 15 randomised trials [15, 16, 37–49]. The forest plot (Fig. 3) shows a standardised mean difference of 0.34 (95% CI 0.18 to 0.50) between intervention and control groups, indicating statistical significance with a small to medium summary effect size [50]. For participants exposed to digital food communication, the overall effect suggests an impact on their behavioural responses to food and nutrition that is consistent with the hypothesis for each intervention (e.g., increases in fruit and vegetable consumption for interventions that targeted increased consumption of fruit and vegetables).

#### Narrative synthesis of randomised trials

Two randomised trials [51, 52] that focused on food consumption did not report outcome data in a manner that could be converted to standardised mean differences for inclusion in the meta-analysis. Both studies confirmed the study hypothesis and showed statistically significant findings (*p* < 0.05). Individual findings from both studies, representing 1,058 participants, are located in the supplementary materials (Table 6).

#### Observational studies on behavioural outcomes

Nine observational studies were identified, reporting on 22 behavioural responses [42, 53–60]. Food consumption accounted for 19 responses and food purchase accounted for three responses. Nineteen responses confirmed the study hypothesis, while three did not. The statistical significance of *p* < 0.05 was used to calculate that eight responses achieved positive significance; ten did not achieve positive significant findings, and significance could not be calculated for three studies due to insufficient data. Individual findings from observational studies, representing 3,505 participants, are in the supplementary materials (Table 7).

### Cognitive outcomes

Twelve studies, representing 2,034 young adults, provided data on cognitive responses. The outcomes assessed intention to consume, purchase intention, and attitudes towards food. These captured explicit and implicit cognitive responses, including intentions to consume specific foods (e.g., sugar-sweetened beverages, multivitamins) and attitudes towards food items (e.g., multivitamins, cheese). Most studies aimed to promote positive cognitive change, such as increased intention to consume nutrient-rich foods or improved attitudes toward healthy eating. One study assessed a negative shift in intention, specifically a reduction in intention to consume sugar-sweetened beverages. In our meta-analysis, the direction of effect was coded as positive [1] for studies promoting healthier food-related cognitions and negative (−1) for studies targeting reductions in intentions toward unhealthy foods. Details on how cognitive responses were assessed in each study are included in the supplementary materials.

#### Meta-analysis

**Fig. 4:**
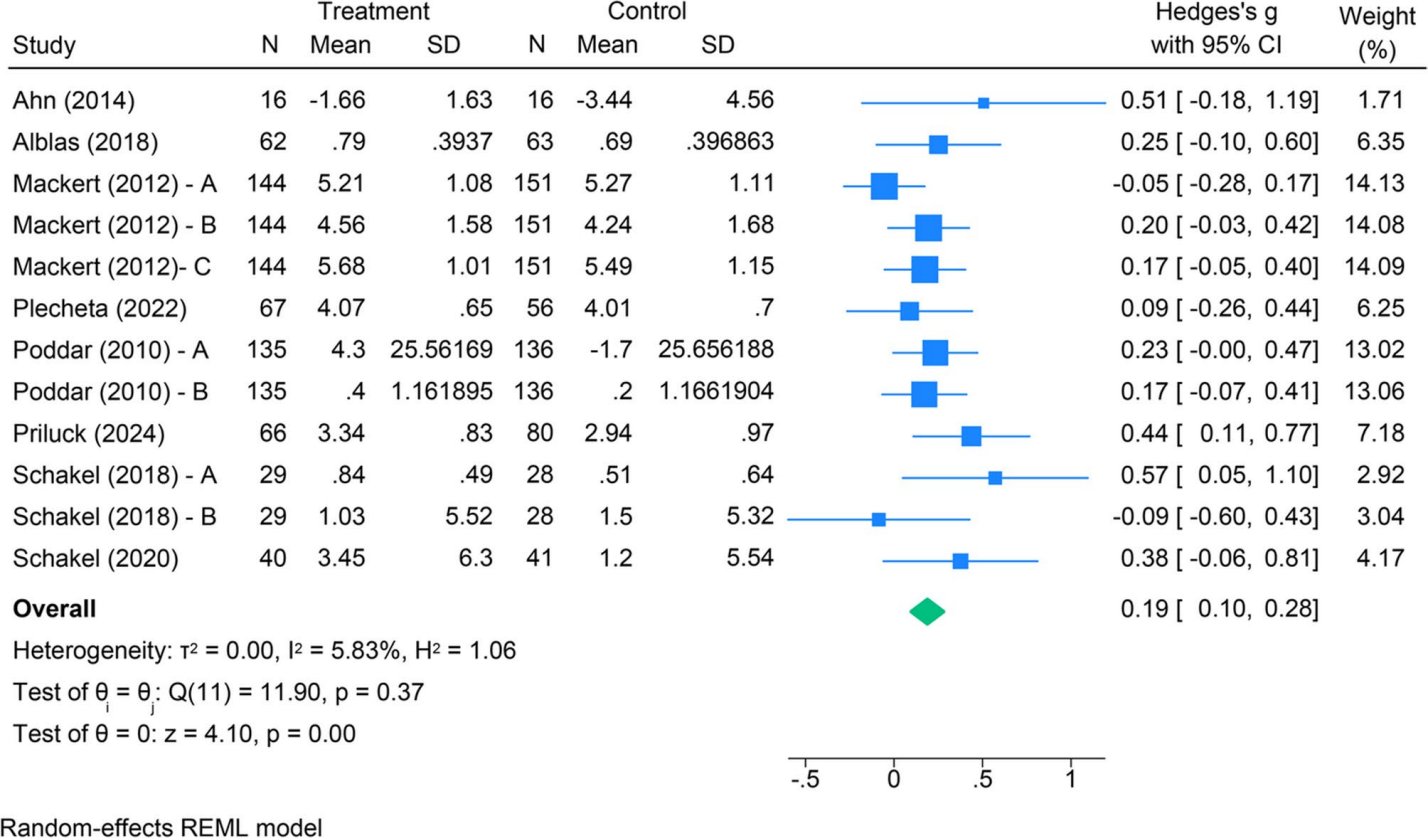
Impact of digital food communication on cognitive responses to food and nutrition

The random-effects meta-analysis of cognitive responses included 1,130 participants from five randomised trials [14, 15, 39, 49, 61–64]. The forest plot (Fig. 4) shows a standardised mean difference of 0.19 (95% CI 0.06 to 0.30) between intervention and control groups, indicating statistical significance with a small summary effect size. For participants exposed to digital food communication, the overall effect favours an impact on their cognitive responses to food and nutrition.

#### Narrative synthesis of randomised trials

One randomised trial that focused on the intention to consume [65] did not report outcome data in a manner that could be converted to standardised mean differences for inclusion in the meta-analysis. The study did not show significant findings, nor did it confirm the study hypothesis. Findings from this study, representing 436 participants, are located in the supplementary materials (Table 8).

#### Observational studies on cognitive outcomes

Four observational studies were identified, reporting on nine cognitive responses [54, 59, 66, 67]. Attitudes towards food accounted for two outcomes, purchase intention accounted for one outcome, and the intention to consume accounted for six outcomes. Eight responses confirmed the study hypothesis of a positive association between digital food communication and cognitive outcomes, while one did not. The statistical significance of *p* < 0.05 was used to calculate that four responses achieved positive significance, and five did not. Individual findings from observational studies, representing 468 participants, are located in the supplementary materials (Table 9).

### Reporting biases

#### Behavioural outcomes

The funnel plot, located in the supplementary materials (Fig. 1), indicates symmetry. The Egger’s test (*p* = 0.88) showed no evidence of small-study effects or publication bias in the funnel plot. The trim-and-fill analysis also supported the reliability of the meta-analysis findings.

#### Cognitive outcomes

In keeping with recommendations from the Cochrane Handbook for Systematic Reviews of Interventions [68], a funnel plot and Egger’s linear probability test for cognitive responses were not conducted because the meta-analysis included fewer than ten studies.

## Synthesis 2: Qualitative synthesis

### Qualitative synthesis – behavioural outcomes

#### Descriptive themes

Nine studies provided qualitative data on behavioural responses to food and nutrition. The three descriptive themes identified (Table 2) were: (a) young adults employ tactics and tools to eat healthy, (b) food purchases and dietary behaviours are influenced by advertisements, and (c) peers influence dietary behaviours. Quotes used to illustrate and exemplify our findings are in the supplementary materials (Table 10).

**Table 2:** Behavioural themes and subthemes of included qualitative studies

Themes	Subthemes	Studies
Young adults employ tactics and tools to eat healthy	Changed dietary behaviours	[52, 69–71]
	Changed relationships with food	[69, 71]
	Utilising mobile apps to increase vegetable intake	[71]
	Utilising tactics to limit exposure to unhealthy foods	[69, 72]
Food purchases and dietary behaviours are influenced by advertisements	Purchases influenced by advertisements	[13, 72–74]
	Dietary behaviours influenced by advertisements	[72, 74]
	Purchases and dietary behaviours influenced by online promotions	[74]
Peers influence dietary behaviours	Dietary behaviours influenced by peer posts and messages	[13, 73]
	Showcasing health behaviours and food to peers through participation in online food culture	[13, 74]

#### Analytical theme

We used descriptive themes to develop one high-level analytical theme.

1. Young adults grapple with aligning their intentions to actual behaviours.

The descriptive themes can be viewed through the lens of balancing behavioural intentions vs. actual behaviour. While participants express strong intentions to adopt healthier eating habits, actual behaviour can be inconsistent due to various factors such as the influence of advertising, social pressure, and the effectiveness of digital tools in maintaining long-term behaviour change. This theme underscores the challenges in translating positive intentions into sustained behavioural change, the importance of addressing external influences and maintaining self-regulation to bridge the gap between intention and action.

*“I’ve seen the 2 fruit 5 veg ad on Facebook multiple times. Definitely something that I find intriguing and occasionally try to do*,* but not always successfully.”* [72].

*“I may have been ‘persuaded’ [read ‘reminded of my weak will’] to purchase [fast food brand name removed] on several occasions.”* [73].

*I think*,* sometimes when I see those café stuff*,* right*,* I feel like people don’t always go there just to try them*,* but out of social pressure. It’s like everybody’s going there*,* so it’s like I have to go there and try also*,* otherwise I would be left out.* [74]

### Qualitative synthesis - cognitive outcomes

Fourteen studies provided data on cognitive responses to food and nutrition. The six descriptive themes identified (Table 3) were: (a) food choices are influenced by cost, (b) healthy eating information impacts awareness and motivation, (c) digital interventions positively impact eating considerations, (d) peer messages influence dietary considerations, (e) perceptions of food-related marketing and messages, and (f) young adults are suspicious of food promotion strategies and diet advice. Quotes used to illustrate and exemplify our findings are in the supplementary materials (Table 11).

**Table 3:** Cognitive themes and subthemes of included qualitative studies

Themes	SubthemeS	Studies
Food choices are influenced by cost	Cost viewed as a barrier to healthy eating	[72, 75]
	Budget-friendly fast food advertisements influence purchase intention	[72, 73]
Healthy eating information impacts awareness and motivation	Exposure to healthy eating information influences awareness & motivation	[13, 52, 69, 71, 73, 75]
	Exposure to online role models and information motivates healthy eating	[73–75]
	Recipes inspire meal considerations	[13, 71, 76]
	Healthy eating messages impact body perceptions	[3]
Digital interventions positively impact healthy eating considerations	Exposure to virtual foods can change attitudes towards disordered eating	[77]
	Participation in digital intervention improves wellbeing	[52]
Peers influence dietary considerations	Peers influence dietary intentions	[73, 74]
	Peer feedback modulates effect of online content	[74]
	Reassurance derived from social media ‘commenters’ as virtual peers	[70]
Perceptions of food-related marketing and messages	Marketing tactics influence perceptions	[3, 66, 72, 73]
	Fast food ads viewed as negative and annoying	[72, 73]
	Exposure to fast food advertisements disrupt intentions for healthy eating	[72, 73]
	Diet-related messages impact body perceptions	[3, 78]
	Perceptions that healthy eating campaigns/ads do not target young adults and recommendations	[3, 78]
Young adults are suspicious of food promotion strategies and diet advice	Unconvinced by food promotion strategies	[13, 66, 72]
	Distrust of diet-related advice/information	[3, 70, 74–76, 78]
	Budget-friendly fast food ads viewed as deceptive	[72]

#### Analytical themes

We used descriptive themes to develop two high-level analytical themes.

1. Good nutrition is difficult for young adults to understand and navigate.

Young adults obtain nutritional information from digital food communication. Currently, this means they absorb conflicting nutritional information from marketing adverts and content created by real and virtual peers. ‘Fitspiration’ content also links the portrayal of healthy diets to body image, which impacts self-efficacy and message internalisation. Young adults must also grapple with conflicting information about nutrition, which encourages scepticism. In all this, young adults do not feel targeted by Government campaigns and advertisements, leaving them without credible sources for health information. This theme spotlights Government efforts that seemingly fail to engage and inform young adults of good nutrition.

*I don’t think they [Government dietary messages] target us.* [3]

*Regarding how best to deliver nutrition information there was consensus it should be online incorporating social media: “Social media because that’s what we’re exposed to the most – but the dietary guidelines aren’t on social media”.* [3]

*I just find understanding nutrition like really difficult*,* like I just don’t get it.* [76]

*I dislike these [fast food] ads because they are misleading and don’t offer anything positive. Often working class people feel as though these foods are all they can afford*,* due to dollar menus and $5 meals*,* however it is consistently shown that whole foods are cheaper in the end.* [72]

2. Content perception informs its impact on young adults.

A key distinction exists between content that young adults perceive as helpful and engaging and that which is viewed as misleading or counterproductive. This distinction impacts the success of digital food communication interventions and campaigns. Notably, ‘good content’ is not confined to government health messages, nor is ‘negative content’ restricted to ads, even though fast food promotions often exemplify this. ‘Good content’ is valued for its transparency, relevance, and engaging design, whereas ‘negative content’ is criticised for its unrealistic portrayals, misleading claims, repetitive nature, and pessimistic messaging. This theme highlights the importance of engagement and presentation in digital food communication.

*Most ads on Facebook influence my health negatively.as they are usually for unhealthy food options.* [73]

*Health promotion messages are so negative – like this is what happens to your gut when you drink coke. I know it’s supposed to have an impact*,* but I don’t feel like it has a significant impact.* [3]

*I see a lot about healthy lifestyle and fitness in my social media feeds and I think that constant exposure has made me much more conscious of the choice I make*,* and a bit more aware of exercising and eating healthy.* [73]

*“V [V Energy] showed their nutrient contents*,* I was surprised by the low guarana content*,* it is not shady at all*,* really appealing”.* [66]

### Certainty of evidence

A summary of the Confidence in the Evidence from Reviews of Qualitative research (GRADE-CERQUal) assessing evidence relating to each descriptive theme is provided in the supplementary materials (Table 16). There was high confidence in evidence used in the three descriptive themes related to behavioural responses. Five of the six descriptive themes on cognitive responses provided high confidence. There were minor concerns regarding the methodological limitations of one descriptive theme on cognitive responses.

## Synthesis 3: Cross-Study matrix

Drawing from the analytical themes of qualitative studies, we developed 10 recommendations for future digital food communication interventions and campaigns to support healthy eating among young adults. They are:

1. Acknowledge social and financial factors affecting healthy eating and foster open dialogue to empower young adults to develop their own solutions.
  - Consider addressing factors such as peer pressure to consume unhealthy foods, the influence of fast food marketing, and young people’s perceptions of the financial challenges associated with healthy eating.
2. Deliver reminders and nudges to increase consumption of healthy foods, using apps and short messages.
3. Integrate jingles, repetition, and social media trends into healthy food advertisements and campaigns to drive engagement and action.
4. Provide affordable, practical, healthy recipes and outline healthy meal options for eating out.
5. Target and engage young adults in dietary interventions and campaigns by using individuals who they find relatable.
6. Utilise co-design to make digital food communication interventions and campaigns believable and actionable for young adults.
7. Emphasise actionable changes and strategies that young adults can incorporate into their daily lives to improve their diet.
8. Provide incentives to purchase healthy foods.
  - These incentives could mirror the successful implementation of promotional codes and discount vouchers used by food marketing companies.
9. Reduce emphasis on adverse (negative) outcomes associated with eating unhealthy foods.
10. Emphasise the credibility of health information used in digital food communication.

Using the recommendations, we assessed their utilisation in the experimental studies included in the review (Table 4). None of the reviewed experimental studies implemented all the qualitative recommendations, and no individual study incorporated more than two. Among those that did implement two, each applied a unique combination. Three of the five studies that implemented two recommendations [37, 38, 47] used a multi-pronged approach in their digital food communication interventions. However, Kerr et al., [38] had mixed results in the findings on behavioural responses with some confirming study hypotheses and one did not.

**Table 4:** Cross-study synthesis matrix

THEMES AND ASSOCIATED BARRIERS	RECOMMENDATIONS	EXPERIMENTS INCORPORATING RECOMMENDATIONS
***Theme 1*** Young adults grapple with aligning their intentions to actual behaviours Associated barriers & facilitators ♣ Personal intention to eat healthy competes with the influence of peers, financial cost and allure of fast food ads ♣ Fast food marketing impacts purchase and consumption ♣ Digital food communication can support adoption of healthier eating habits and relationships with food ♣ Peers can encourage or detract from healthy eating efforts	1. Acknowledge social and financial factors affecting healthy eating and foster open dialogue to empower young adults to develop their own solutions 2. Deliver reminders and nudges to increase consumption of healthy foods, using apps and short messages 3. Integrate jingles, repetition, and social media trends into healthy food advertisements and campaigns to drive engagement and action	1. None identified 2. [37, 38, 44, 47, 51, 52] 3. None Identified
***Theme 2*** Good nutrition is difficult to understand and navigate. Associated barriers & facilitators ♣ Multifaceted influences of cost, information, peer behaviour and marketing impact the understanding of good nutrition ♣ Portrayal of healthy diets linked to body image which impacts self-efficacy and message internalisation ♣ Scepticism towards dietary-related messages as recommendations seem unrealistic ♣ Perceived cost of healthy food items vs. fast foods makes healthy eating seem unachievable ♣ Young adults don’t feel targeted by Government dietary campaigns or healthy eating ads ♣ Conflicting dietary information from multiple sources	4. Provide affordable, practical, healthy recipes and state healthy meal options for eating out 5. Target and engage young adults in dietary interventions and campaigns by using relatable individuals 6. Utilise co-design to make digital food communication interventions and campaigns believable and actionable for young adults 7. Emphasise actionable changes/strategies that young adults can incorporate in their daily lives to improve their diet 8. Provide financial incentives to purchase healthy foods	4. [38, 45, 48] 5. None Identified 6. [40] 7. [47, 48, 53, 54] 8. None Identified
***Theme 3*** Content perception informs its impact on young adults. Associated barriers & facilitators ♣ Conflicting dietary information impact intention and behaviour ♣ ‘Good content’ is valued for its transparency, relevance, and engaging design ♣ ‘Negative content’ criticised for unrealistic portrayals, misleading claims, repetitive nature, and pessimistic messaging. ♣ Dietary recommendations viewed as pessimistic or unachievable ♣ Brands sometimes commended for transparency	9. Reduce emphasis on adverse (negative) outcomes associated with eating unhealthy foods 10. Emphasise the credibility of health information used in digital food communication	9. [54, 61] 10. [37, 40, 41]

The recommendations to target and engage young adults in dietary interventions and campaigns by using relatable individuals and providing financial incentives to purchase healthy foods were not implemented in any studies. Additionally, no study acknowledged and sought to mitigate both the (possible) impacts of peers and financial wellbeing on the outcome of a digital food communication intervention or experiment. However, several studies [42, 54, 55, 57, 59] sought to replicate elements of peer/social interaction in their interventions.

The included studies varied in their aims, target behaviours, and modes of delivery. While no study implemented more than two recommendations, this may reflect a selective approach based on study design, feasibility, or population focus, rather than a lack of alignment with best practices. Our intention in presenting this synthesis is not to imply that a higher number of recommendations equates to better design, but rather to highlight opportunities where future interventions might be strengthened by drawing on multiple, context-appropriate strategies.

## Discussion

This systematic review evaluates the impact of digital food communication on behaviours and cognitive responses among young adults. Meta-analyses of randomised trials on behaviours and cognitive responses demonstrated significance with small to medium summary effect sizes SMD of 0.34 (95% CI: 0.18–0.50) and 0.19 (95% CI: 0.10–0.28), respectively. Observational studies underscored the findings of the meta-analyses, with most individual studies confirming a change in behaviours and cognitive responses, even when not statistically significant. Difficulty deciphering what represents good nutrition, critical distinctions when engaging with content perceived as helpful vs. misleading and balancing healthy eating intentions against actual behaviours represented barriers to the effectiveness of digital food communication. Ten recommendations were developed to support future digital food communication interventions and campaigns on healthy eating, targeting young adults. We also assessed the utilisation of these recommendations by experimental studies in the review, finding that no individual study implemented more than two recommendations, suggesting that current experimental approaches only partially reflect the full range of strategies identified by young adults as relevant.

The study offers an overarching look at digital food communication, building on systematic reviews that concentrate on a single platform, namely social media [79, 80], video games [81] and virtual reality [82]. The review also benefits from qualitative studies, shedding light on the connection between digital food communication and its potential to cause positive and negative health outcomes; a topic explored in a recent systematic review [83] looking at the positive and negative impact of social media influencers on health outcomes.

The findings from the analytical themes and the cross-study matrix, including the ten recommendations developed, highlight the value of a holistic approach when engaging young adults about healthy eating using digital food communication. Story et al. [84] advocates for a similar approach in their ‘conceptual framework for understanding adolescent eating behaviours’, which includes individual factors and environmental influences, namely socio-cultural changes, family and the school environment. However, there are important differences for young adults: The increased independence and responsibility for nutrition, a reduced role of the family in dietary considerations, financial burdens, distrust of information sources about nutrition and lack of clarity about what represents good nutrition.

A key strength of this review is the mixed-methods approach. Mixed-method studies benefit from “integrating the power of stories and the power of numbers [to] compensate for their respective limitations” [52]. This approach allows the review to provide a comprehensive look at the impact of digital food communication on young adults’ behaviours and cognitive responses towards food and nutrition. As digital food communication covers several disciplines, consultations with a health librarian and tailoring the Boolean search to different databases added rigour to the search strategy. The review also benefitted from the academic and language diversity of the team screening articles and extracting data, allowing for the evaluation of articles published in English and Spanish from different disciplines.

While we used databases that contain articles from across the globe, the included studies are from seventeen countries, all categorised as high-income and upper-middle-income, limiting applicability to low-income and lower-middle-income countries. We, therefore, could not conduct the planned subgroup analysis comparing findings from high-income vs. middle/low-income countries.

We included studies where the median or average age is between 18 and 25, ensuring we captured relevant studies for young adults. However, this means that not all the included studies were focused exclusively on young adults.

Another limitation is that the majority of studies in this review focused on university and college students, which may limit generalisability to the broader young adult population, particularly those not engaged in higher education. Additionally, a key limitation of systematic reviews is their reliance on information in the published articles; the evaluation of whether studies incorporated recommendations was, therefore, based on details of the published articles and may not fully reflect the implementation details or intentions of the original authors.

We considered that the effect size for Liu [44] was large and ran the meta-analysis of the behavioural outcomes without it, and the findings remained statistically significant. Methodological limitations were identified: the mixture of low, medium, and high-quality studies in the review and the use of qualitative data collected and interpreted by original study authors with different ontological and epistemological approaches. However, the evidence remains consistent with the impact of digital food communication on young adults’ behaviours and cognitive responses to food and nutrition.

The impact of digital food communication on food purchase and consumption, and attitudes towards food and nutrition encourage researchers and policymakers to approach digital food communication as a digital determinant of the dietary health for young adults. The findings echo calls for a shift in health policy to engage young adults using individual and population-level dietary consumption policies [8, 85]. Our recommendations for digital food communication interventions and campaigns to support healthy eating offer actionable, user-informed steps that policymakers and practitioners can apply to design more engaging and relevant strategies for young adults.

The review adds evidence to the discourse on the food industry’s role in health outcomes [86–88]. Young adults expressed mixed views on the role of the food industry. Fast food marketing adverts and promotional tactics derail efforts to eat healthily. At the same time, they appreciated its provision of ‘affordable’ meal options. The findings, therefore, offer another opportunity to consider the importance of regulating fast food advertisements online and the need to provide cost-effective, healthy food options for young adults, limiting their need to turn to unhealthy foods.

The review accepts the complex nature of young adults’ behaviours and cognitive responses that rely on personal willpower as much as environmental, financial, and social realities. However, several recommendations were not implemented in the quantitative studies in the review, namely, using relatable individuals to support targeted digital food communication, providing financial incentives to purchase healthy foods, and mitigating the (possible) impacts of peers and financial well-being on dietary change. This lack of implementation allows researchers to expand the variables considered when conducting studies on the influence of digital food communication on behaviours.

Finally, the review illustrates the need to research emerging areas where digital food communication is leveraged to impact young adults through food-related videos, live-streaming platforms, and artificial intelligence. It may also be time to conduct multi-country studies that consider the experiences of young adults with digital food communication in regions not represented in this review, such as Central America, the Caribbean and the Pacific.

## Conclusions

This review of 45 studies, published between 2011 and 2024, represents 8,914 young adults across 16 countries. The findings provide strong evidence that digital food communication influences young adults’ cognitive and behavioural responses to food and nutrition. It also highlights the barriers to healthy eating and offers recommendations for future digital food communication interventions and campaigns aimed at promoting healthier eating habits.

## Supplementary Information

Supplementary Material 1

Supplementary Material 2

Supplementary Material 3

## Data Availability

No datasets were generated or analysed during the current study.

## Abbreviations

CASP: Critical Appraisal Skills Programme
GRADE- CERQUal: Grading of Recommendations, Assessment, Development and Evaluation – Confidence in Evidence from Reviews of Qualitative Research
PROSPERO: International prospective register of systematic reviews
S.P.I.D.E.R: Sample, Phenomenon of Interest, Design, Evaluation, and Research-type
