# Supplementary material for: Behavioural and cognitive changes in young adults towards food and nutrition after exposure to digital food communication: a mixed-methods systematic review
Source: Int J Behav Nutr Phys Act. 2025 Oct 20;22:128. doi: 10.1186/s12966-025-01817-5 (PMC12538972; doi:10.1186/s12966-025-01817-5)
Supplement: Supplementary file 3 — Supplementary Material 3 [file 12966_2025_1817_MOESM3_ESM.docx]

**Supplementary Materials**

Behavioural and cognitive changes in young adults towards food and nutrition after exposure to digital food communication: a mixed-methods systematic review

Contents

[**Table 1:** S.P.I.D.E.R. tool 3](#_Toc204211543)

[**Table 2:** Boolean search by academic database 4](#_Toc204211544)

[**Table 3**: Characteristics of Included Studies - Quantitative Articles (N=33) 6](#_Toc204211545)

[**Table 4**: Characteristics of Included Studies - Qualitative Articles (N= 14) 9](#_Toc204211546)

[**Table 5**: Articles excluded at full screening 10](#_Toc204211547)

[**Table 6**: Experimental Studies – Behavioural (Narratives of Outcome Data) 17](#_Toc204211548)

[**Table 7**: Observational Studies – Behavioural (Narratives of Outcomes Data) 17](#_Toc204211549)

[**Table 8:** Experimental Studies – Cognitive (Narratives of Outcomes Data) 23](#_Toc204211550)

[**Table 9**: Observational Studies – Cognitive (Narratives of Outcomes Data) 23](#_Toc204211551)

[**Table 10**: Behavioural Themes, Subthemes and Quotes 25](#_Toc204211552)

[**Table 11**: Cognitive Themes, Subthemes and Quotes 32](#_Toc204211553)

[**Table 12**: Risk of Bias Assessment – ROB1 46](#_Toc204211554)

[**Table 13**: Risk of Bias Assessment – JBI Quasi-Experiment Checklist 48](#_Toc204211555)

[**Table 14**: Risk of Bias Assessment – Newcastle Ottawa Scale 49](#_Toc204211556)

[**Table 15**: Risk of Bias Assessment – Critical Appraisal Skills Programme (CASP) 50](#_Toc204211557)

[**Table 16**: GRADE-CERQUal summarised review of descriptive findings 51](#_Toc204211558)

[**Fig. 1**: Assessment of publication bias of studies focused on behavioural responses in meta-analysis 53](#_Toc204211559)

## **Table 1:** S.P.I.D.E.R. tool

| S.P.I.D.E.R. | Review definitions |
| --- | --- |
| SAMPLE | Young adults aged 18 - 25 years |
| PHENOMENA OF INTEREST | Digital food communication which occurs on social media, interactive media and the internet by leveraging digital marketing, influencer marketing or influencer engagement, digital food communities, blogs, websites and activities by “ordinary people as participants in online food culture” (Lewis, 2018). |
| DESIGN | All primary qualitative, mixed methods and quantitative research, published in peer-reviewed journals and written in English or Spanish.   - Quantitative methods (e.g.: experimental studies, longitudinal studies, cross sectional studies) - Qualitative methods (e.g.: case studies, ethnography, interviews, focus groups) - Mixed method studies: Hong et al (2017) defines mixed method studies as “research involving collecting and integrating both quantitative and qualitative data.” |
| EVALUATION | - Behavioural response is a response reflecting the implicit and/or explicit response of the body   (i.e.: food consumption and food purchase)   - Cognitive responses are explicit opinions, choices and decisions involving cognitive processing, which may be unknowingly influenced by an implicit response   (i.e.: intention to consume, intention to purchase and attitudes towards food and nutrition) |
| RESEARCH TYPE | - Quantitative - Qualitative - Mixed Methods |

## **Table 2:** Boolean search by academic database

|  | SEARCH TERMS | PsycINFO | BUSINESS SOURCE ULTIMATE | PubMed | GLOBAL INDEX MEDICUS |
| --- | --- | --- | --- | --- | --- |
| 1 | Food | (food or nutrition* or diet* or meal* or drink* or beverage* or vegan* or vegetarian* or "plant based" or fruit* or vegetable* or nuts or fiber or fibre).mp. | (food or nutrition* or diet* or meal* or drink* or beverage* or vegan* or vegetarian* or "plant based" or fruit* or vegetable* or nuts or fiber or fibre) | food[Text Word] OR nutrition*[Text Word] OR diet*[Text Word] OR meal*[Text Word] OR drink*[Text Word] OR beverage*[Text Word] OR vegan*[Text Word] OR vegetarian*[Text Word] OR "plant based"[Text Word] OR fruit*[Text Word] OR vegetable*[Text Word] OR nuts[Text Word] OR fiber[Text Word] OR fibre[Text Word] | (food OR nutrition* OR diet* OR meal* OR drink* OR beverage* OR vegan* OR vegetarian* OR "plant based" OR fruit* OR vegetable* OR nuts OR fiber or fibre) AND ("young men*" OR "young males" OR "young adult" OR "young person" OR youth* OR millennial* OR "Generation Z" OR "young generation" OR "younger generation*" OR "young women" OR "young females" OR "younger population" OR "student*") AND **(Facebook or Twitter or YouTube or Google or Snapchat or Pinterest or WhatsApp or Reddit or YouTuber or fitspiration or thinspiration or Tumblr or hashtag* or newsfeed* or "web 2.0" or blog* or vlogs* online communit* or food communit* or website* or "web based intervention*" or influencer* or tweet* or Instagram* or tiktoker* or "tik tok*" or "social media" or social network or "algorithmic marketing" or "algorithmic advertising" or "online trend*" or mobile application* or podcast* or virtual reality or video game* or "internet trend*" or interactive storytelling or augmented reality or ((Marketing or "health communication" or advertising) and (online or "web-based" or digital* or internet)))** |
| 2 | Population | ("young men*" or "young male*" or "young adult*" or "young person" or youth* or millennial* or "Generation Z" or "young generation" or "younger generation*" or "young women" or "young female*" or "younger population*" or "student*"). mp. | ("young men*" or "young male*" or "young adult*" or "young person" or youth* or millennial* or "Generation Z" or "young generation" or "younger generation*" or "young women" or "young female*" or "younger population*" or "student*") | "young men*"[Text Word] OR "young male*"[Text Word] OR "young adult*"[Text Word] OR "young person*"[Text Word] OR youth*[Text Word] OR millennial*[Text Word] OR "Generation Z"[Text Word] OR "young generation*"[Text Word] OR "younger generation*"[Text Word] OR "young women"[Text Word] OR "young female*"[Text Word] OR "younger population*"[Text Word] OR "student*"[Text Word] |  |
| 3 | Digital Food Communication | (Facebook or Twitter or YouTube or Google or Snapchat or Pinterest or WhatsApp or Reddit or YouTuber or fitspiration or thinspiration or Tumblr or hashtag* or newsfeed* or "web 2.0" or blog* or vlog* or online communit* or food communit* or website* or "web based intervention*" or influencer* or tweet* or Instagram* or tiktoker* or "tik tok*" or "social media" or social network or "algorithmic marketing" or "algorithmic advertising" or "online trend*" or mobile application* or podcast* or virtual reality or video game* or "internet trend*" or interactive storytelling or augmented reality or ((Marketing or "health communication" or advertising) and (online or "web-based" or digital* or internet))).mp. | (Facebook or Twitter or YouTube or Google or Snapchat or Pinterest or WhatsApp or Reddit or YouTuber or fitspiration or thinspiration or Tumblr or hashtag* or newsfeed* or "web 2.0" or blog* or vlog* or online communit* or food communit* or website* or "web based intervention*" or influencer* or tweet* or Instagram* or tiktoker* or "tik tok*" or "social media" or social network or "algorithmic marketing" or "algorithmic advertising" or "online trend*" or mobile application* or podcast* or virtual reality or video game* or "internet trend*" or interactive storytelling or augmented reality or ((Marketing or "health communication" or advertising) and (online or "web-based" or digital* or internet))) | **Facebook[Text Word] OR Twitter[Text Word] OR YouTube[Text Word] Google[Text Word] OR Snapchat[Text Word] OR Pinterest[Text Word] OR WhatsApp[Text Word] OR Reddit[Text Word] OR YouTuber[Text Word]OR fitspiration[Text Word] OR thinspiration[Text Word] OR Tumblr[Text Word] OR hashtag*[Text Word] OR newsfeed*[Text Word] OR "web 2.0"[Text Word] OR blog[Text Word] OR vlog* [Text Word] OR online communit*[Text Word] OR Website*[Text Word] OR "web based intervention*"[Text Word] OR influencer*[Text Word] OR tweet*[Text Word] OR Instagram*[Text Word] OR tiktoker*[Text Word] OR "tik tok*"[Text Word] OR "social media"[Text Word] OR “social network” [Text Word] OR "algorithmic marketing"[Text Word] OR "algorithmic advertising"[Text Word] OR "online trend*"[Text Word] OR mobile application*[Text Word] OR podcast[Text Word] OR Virtual reality[Text Word] OR video game*[Text Word] OR interactive storytelling[Text Word] OR Augmented reality[Text Word] OR "internet trend*"[Text Word] OR ((Marketing[Text Word] OR "health communication"[Text Word] OR advertising[Text Word]) AND (online[Text Word] OR "web-based"[Text Word] OR digital*[Text Word] OR internet[Text Word]))** |  |

## **Table 3**: Characteristics of Included Studies - Quantitative Articles (N=33)

| # | STUDY ID | COUNTRY | STUDY DESIGN | POPULATION & MEAN AGE | SAMPLE SIZE | GENDER | SETTING | DIGITAL MEDIA | OUTCOME (S) |
| --- | --- | --- | --- | --- | --- | --- | --- | --- | --- |
|  | Ahn (2015) | U.S. A | Randomised trial | College students [M=20.83] | 86 | Male & Female | University – IVE station | Virtual reality | Behavioural – Food consumption;  Cognitive – intention to consume |
|  | Alblas (2018) | Netherlands | Randomised trial | College students  [M= 20.17] | 125 | Male & Female | University – Social sciences lab | Video game | Behavioural – Food consumption |
|  | Ashton (2017) | Australia | Randomised trial | Aged 18 - 25  [M = 22.1] | 50 | Male | University, lab and online | Social media; website; wearable device | Behavioural – Food consumption |
|  | Buchanan (2017) | Australia | Randomised trial | Aged 18-24 [M = 20] | 60 | Male & Female | Community – meeting rooms, cafes, parks | Social media; website | Cognitive – Purchase intention, attitudes towards food |
|  | Buchanan (2018) | Australia | Cross sectional | Aged 18-24 years | 359 |  | Online | Social media | Cognitive – intention to consume |
|  | Chung (2021) | Hong Kong (China) | Controlled-experimental study | Aged 18-31 in Hong Kong  [M = 22.1] | 305 | Male & Female | Community, university and online | Mobile apps, social media, wearable device | Behavioural – Food consumption |
|  | Coccia (2020) | U.S. A | Quasi-Experiment | Student athletes [M= 19.62] | 50 | Male & Female | Online | Social media | Behavioural – Food consumption; Cognitive – intention to consume |
|  | Duan (2017) | China | Randomised trial | Undergrad students [M =19] | 493 | Male & Female | University; online | Social media; website | Behavioural – Food consumption |
|  | Duan (2022) | China | Randomised trial (Mixed methods) | College students [M= 19.99] | 552 | Male & Female | University; online | Social media; website | Behavioural – Food consumption |
|  | Goodman (2016) | Canada | Randomised trial | Adults in Ontario aged 18 - 25 | 90 | Male & Female | Online | Mobile apps | Behavioural – Food consumption |
|  | Hawkins (2021) | United Kingdom | Experiment | College students [M=20] | 169 | Female | University; lab | Social media | Behavioural – Food consumption |
|  | Heikkilä (2019) | Finland | Randomised trial | Endurance Athletes [M = 18] | 79 | Male & Female | University; online | Mobile apps | Behavioural – Food consumption |
|  | Hilkens (2021) | Netherlands | Cross sectional | Male gym users  [M= 24] | 2269 | Male | online | Social media | Behavioural – Food consumption |
|  | Kerr (2016) | Australia | Randomised trial | People from 57 suburbs in Perth metropolitan area aged 18-30  [Intervention M = 24.2;  Control M = 25] | 247 | Male & Female | discussion | Mobile apps, website, SMS messages | Behavioural – Food consumption |
|  | Kilb (2023) | Germany | Randomised trial | 18–29 years  [M = 22] | 81 |  | University | Social media | Behavioural – Food consumption |
|  | Kinkel-Ram (2022) | U.S.A | Randomised trial | University students  Site 1; M = 18:59  Site 2; M = 23.70 | Site 1 = 222; Site 2 = 214 | Female | University | Wearable device | Cognitive – Intention to consume |
|  | Krishnamohan (2017) | India | Non  randomised trial | University students aged 18-23 | 45 | Male & Female | University; online | Social media | Behavioural – Food consumption |
|  | Lai (2023) | Taiwan | Randomised trial | University students  [M = 19.88] | 98 | Male & Female | Online | Website (Online course) | Behavioural – Food consumption |
|  | Liu (2024) | China | Randomised trial | University students aged 18-24 | 300 | Male & Female | University; online | Mobile app | Behavioural – Food consumption |
|  | Mackert (2012) | U.S. A | Randomised trial | Female undergraduate students aged 18-24 | 295 | Female | University; online | Social media; website | Cognitive – Intention to consume; attitudes towards food |
|  | Monroe (2015) | U.S.A | Randomised trial | University students [M = 18] | 491 | Male & Female | University | Website | Behavioural – Food consumption |
|  | Mota (2023) | Canada | Randomised trial | University students aged 17-30  [M = 18 & 19] | 98 | Male & Female | Online | Mobile app | Behavioural – Food consumption |
|  | O'Brien (2016) | U.S.A | Randomised trial | University students   [M = 19.2] | 154 | Male & Female | University | Website, SMS messages | Behavioural – Food consumption |
|  | Park (2017) | South Korea | Randomised trial | College women with low bone mass [Control M = 23; Intervention M =24] | 82 | Female | University; online | Mobile app | Behavioural – food consumption |
|  | Plechatá (2022) | Denmark | Randomised trial | University students  [M = 25] | 123 | Male & Female | University | Virtual reality | Behavioural – Food consumption;  Cognitive – Intention to consume |
|  | Poddar (2010) | U.S. A | Randomised trial | University students  [M = 20.2] | 294 | Male & Female | University; online course | Website, email | Cognitive – intention to consume |
|  | Pollack (2022) |  | Cross sectional | Twitch users  [Median = 21] | 568 | Male & Female | Online | Social media | Behavioural – food purchasing |
|  | Priluck (2023) | U.S. A | Randomised trial | Young adults aged 18-22 | 148 |  | Online | Mobile gaming app | Cognitive – attitudes towards food |
|  | Schakel (2018) | Netherlands | Randomised trial | Aged 18-35   [M = 21.3] | 120 | Male & Female | Online; lab | Video games | Behavioural – Food consumption;  Cognitive – attitudes towards food |
|  | Schakel (2020) | Netherlands | Randomised trial | University students 18-35 years [M = 21.9] | 81 | Male & Female | Online | Video games | Cognitive – attitudes towards food |
|  | Sharps (2019) | United Kingdom | Non  randomised trial | Undergrad students [M = 19] | 20 | Male & Female | Online | Social media | Behavioural – food consumption;  Cognitive – intention to consume |
|  | Slazus (2022) | South Africa | Non  randomised  trial | College students aged 18 - 25 | Phase 2 = 61 | Male & Female | Online | Mobile app | Behavioural – food consumption |
|  | Whatnall (2019) | Australia | Randomised trial | University students aged 17-35  [M = 22.4] | 124 | Male & Female | Online | Website | Behavioural – Food consumption; |

## **Table 4**: Characteristics of Included Studies - Qualitative Articles (N= 14)

| # | STUDY ID | COUNTRY | STUDY DESIGN | POPULATION & MEAN AGE | SAMPLE | GENDER | SETTING | DIGITAL MEDIA | OUTCOME (S) |
| --- | --- | --- | --- | --- | --- | --- | --- | --- | --- |
|  | Brennan (2020) | Australia | Digital Ethnography | Aged 18 - 25 | 195 | Male & Female | Online | Social media | Behavioural; Cognitive |
|  | Buchanan (2017) | Australia | Mixed methods: Non-randomised trial; semi-structured interviews | Aged 18-24 | 60 | Male & Female | Community; online | Social media; website | Behavioural; cognitive |
|  | Cardi (2012) | United Kingdom | Case Report | Aged 21 | 1 | Female | Clinic | Virtual reality | Cognitive |
|  | Duan (2022) | China | Mixed methods: Randomised Trial; interviews | College students [M= 19.99] | 552 | Male & Female | University; online | Social media; website | Behavioural |
|  | Easton (2018) | United Kingdom | Interviews; focus groups | Fitspiration followers aged 18 - 25  [M = 20.7] | 20 | Male & Female |  | Social media | Behavioural; cognitive |
|  | Friedman (2022) | Australia | Digital ethnography | Aged 18-24 | 234 | Male & Female | Online | Social media | Behavioural; cognitive |
|  | Lambert (2019) | Australia | Focus groups | University students aged 18 - 25 | 49 | Male & Female | University | Social media | Cognitive – attitudes towards food |
|  | Leu (2022) | Singapore | Focus groups | Students aged 19-24 | 46 | Male & Female | Polytechnic and university | Social media | Behavioural; cognitive |
|  | Malloy (2024) | New Zealand | End-User Workshops | Young women aged 18-24 | 19 | Female | University | Social media | Cognitive |
|  | Molenaar (2020) | Australia | Digital ethnography | Aged 18-24 | 166 | Male & Female | Online | Social media | Behavioural; cognitive |
|  | Molenaar (2021) | Australia | Digital ethnography | Aged 18-24 | 166 | Male & Female | Online | Social media | Behavioural; cognitive |
|  | Nour (2019) | Australia | Mixed methods: intervention; structured interviews | M = 24.8 | 97 | Male & Female | Online | Social media; mobile applications | Behavioural; cognitive |
|  | Vaterlaus (2015) | U.S. A | Focus groups; semi-structured interviews | College students aged 18 - 25 [M= 20.4] | 34 | Male & Female | University | Social media | Behavioural; cognitive |
|  | Vedovato (2021) | Brazil | Intervention | College students [M=23] | 82 |  | University | Website | Behavioural; cognitive |

## **Table 5**: Articles excluded at full screening

| **#** | **STUDY ID** | **STUDY TITLE** | **REASON FOR EXCLUSION** |
| --- | --- | --- | --- |
|  | Ahn (2016) | Virtual exemplars in health promotion campaigns: Heightening perceived risk and involvement to reduce soft drink consumption in young adults. | Study design – control and intervention used digital food communication |
|  | Al-Bisher (2022) | Eating Concerns Associated with Nutritional Information  Obtained from Social Media among Saudi Young  Females: A Cross-Sectional Study | Study design |
|  | Ali (2021) | Feasibility Study of a Newly Developed Technology-Mediated Lifestyle Intervention for Overweight and Obese Young Adults. | Study design – control and intervention used digital food communication |
|  | Aljefree (2022) | Exposure to Food Marketing via Social Media and Obesity among University Students in Saudi Arabia. | Population – No mean, median or average age provided |
|  | Allman-Farinelli (2019) | The Role of Supportive Food Environments to Enable Healthier Choices When Eating Meals Prepared Outside the Home: Findings from Focus Groups of 18 to 30-Year-Olds. | Population – no mean age provided |
|  | Almenera (2019) | Sociodemographic, attitudinal, and behavioral correlates of using nutrition, weight loss, and fitness websites: An online survey. | Data presentation – could not pull data from what was presented |
|  | Ashton (2023) | Exploring the design and utility of an integrated web-based chatbot for young  adults to support healthy eating: a qualitative study | Study design – participant discussions focused on improving chatbots |
|  | Ashraf (2022) | Effects of Social Media Apps on University Students’ Health Behaviour in Physical Activity, Weight Loss, and Nutrition Perspective. | Population – no mean, average of median age of all participants provided |
|  | Bangia (2017) | A Point-of-Purchase Intervention Using Grocery Store Tour Podcasts About Omega-3s Increases Long-Term Purchases of Omega-3-Rich Food Items. | Population – mean age = 50.4 |
|  | Barchitta (2022) | Development of a Web-App for the Ecological Momentary Assessment of Dietary Habits among College Students: The HEALTHY-UNICT Project. | Outcomes – tested validity of web app; not impact on behavioural or cognitive outcomes |
|  | Bathgate (2017) | Feasibility of Assessing Diet with a Mobile Food Record for Adolescents and Young Adults with Down Syndrome | Exposure – food data inputted using technology, but participants did not receive any information |
|  | Bejar (2022) | Evaluation of an Application for Mobile Telephones (e-12HR) to Increase Adherence to the Mediterranean Diet in University Students: A Controlled, Randomized and Multicentric Study | Study design – control and intervention used digital food communication |
|  | Bejar (2023) | Effectiveness of a Smartphone App (e-12HR) in Improving Adherence to the Mediterranean Diet in Spanish University Students by Age, Gender, Field of Study, and Body Mass Index: A Randomized Controlled Trial. | Study design – control and intervention used digital food communication |
|  | Brennan (2010) | Persuading young consumers to make healthy nutritional decisions. | Population – no average, mean or median age provided |
|  | Brown (2011) | Increased Self-efficacy for Vegetable Preparation Following an Online, Skill-based Intervention and In-Class Tasting Experience as Part of a General Education College Nutrition Course | Study design |
|  | Browne (2019) | You wouldn't eat 16 teaspoons of sugar-so why drink it? Aboriginal and Torres Strait Islander responses to the LiveLighter sugary drink campaign. | Population – no average, mean or median age provided |
|  | Bramlett (2012) | Safe Eats: an evaluation of the use of social media for food safety education | Study design |
|  | Cai (2023) | Effect of WeChat-based intervention on food safety knowledge, attitudes and practices among university students in Chongqing, China: a quasi-experimental study | Study design |
|  | Chung (2017) | Tweeting to Health: A Novel mHealth Intervention Using Fitbits and Twitter to Foster Healthy Lifestyles | Data presentation – Last pre and post data on fruit and vegetable intake. |
|  | Cousineau (2006) | Web-based nutrition education for college students: Is it feasible? | Outcomes – focus on obtaining feedback on a web-based programme; not impact of digital food communication |
|  | De Grove (2019) | Okay to promote eating less meat, but don’t cheat – The role of dietary identity, perceived inconsistency and inclusive language of an advocate legitimizing meat reduction | Study design – lacks connection between digital food communication and behavioural or cognitive outcomes |
|  | Dickinson (2013) | Use of a website to accomplish health behavior change: if you build it, will they come? And will it work if they do? | Population – mean age = 42.5 |
|  | Doub (2016) | Identifying users of traditional and internet-based resources for meal ideas: An association rule learning approach. | Population – 68% of population over 35 |
|  | Drivas (2024) | #WhatIEatInADay: The effects of viewing food diary TikTok videos on young adults' body image and intent to diet. | Outcome – study doesn’t test whether there is a direct relationship between exposure and intention to diet; only looks at a mediated relationship |
|  | Duren-Winfield (2021) | The Development and Pilot Testing of an Evidence-Based Cardiovascular Disease Risk-Prevention Program to Promote Healthy Lifestyle Behaviours Among African-American College Students – Rams have HEART | Study design – no data on relevant cognitive and behavioural outcomes |
|  | Espedido (2021) | I Read It on Reddit: Food Safety Information-Seeking Preferences and Practices of Young Adults Online. | Population – participants were 18-39. No mean or average age provided |
|  | Fackler (2021) | Technology-Enhanced Health Promotion for College Students: A Seed Development Project. | Population – age of participants not provided |
|  | Ferrer-Garcia (2014) | Validation of VR-based Software for Binge Eating Treatment: Preliminary Data. | Population – no age provided |
|  | Filippone (2022) | The relationships between social media exposure, food craving, cognitive impulsivity and cognitive restraint. | Population – Mean age – 29.32 |
|  | Folkvord (2020) | Promoting healthy foods in the new digital era on Instagram: an experimental study on the effect of a popular real versus fictitious fit influencer on brand attitude and purchase intentions. | Study design - looks at the parasocial link between real vs fictitious influencer; not the impact of digital food communication |
|  | Forman (2019) | OnTrack: Development and feasibility of a smartphone app designed to predict and prevent dietary lapses. | Population – wrong age group |
|  | Frederick (2022) | Millennials Purchase Intention based on Food Vlogger Reviews - A Micro Study at Palghar District, Maharashtra. | Population – no mean, average or median age provided |
|  | Garzon Medina (2021) | Influence of advertising on social media on emotional response and purchase intention of functional dairy beverages | Population – participants were aged 18-35. No mean, average or median age provided |
|  | Gleaves (2024) | I'll have what She's having (but not what They're having): The moderating role of group membership in the effect of social norms on food choice in an online environment. | Study design - control and intervention used digital food communication |
|  | Hahn (2021) | Introducing Dietary Self-Monitoring to Undergraduate Women via a Calorie Counting App Has No Effect on Mental Health or Health Behaviors: Results from a Randomized Controlled Trial. | Study design – participants did not receive any information via digital food communication but only inputted dietary info on app |
|  | Hammond (2018) | Exposure and perceptions of marketing for caffeinated energy drinks among young Canadians. | Population – no mean age provided for all participants |
|  | Haslam (2023) | Efficacy of technology-based personalised feedback on diet quality in young Australian adults: results for the advice, ideas and motivation for my eating (Aim4Me) randomised controlled trial. | Study design – intervention and control groups used digital food communication |
|  | Hawkins (2020) | Do perceived norms of social media users' eating habits and preferences predict our own food consumption and BMI? | Study design – lack of emphasis on digital food communication |
|  | Hawkins (2024) | Can social media be used to increase fruit and vegetable consumption? A pilot intervention study. | Population - no mean, average or median age provided |
|  | Hubert (2022) | Feasibility of a Theory-Based, Online Tailored Message Program to Motivate Healthier Behaviors in College Women. | Study design – focus on how to target health messages instead of impact of digital food communication |
|  | Inauen (2017) | Using smartphone-based support groups to promote healthy eating in daily life: A randomised trial. | Population – average age of participants is 27 years |
|  | Jandrokovic (2022) | Analysis of Influencer Marketing in the Positioning Healthy Eating According to Generation Z in Croatia. | Population – No mean, median or average age provided |
|  | Jayanetti (2018) | Pizza, burgers and booze: online marketing and promotion of food and drink to university students. | Study design – analysis of social media accounts not linked to age group of population |
|  | Jett (2010) | Impact of exposure to pro-eating disorder websites on eating behaviour in college women. | Population – no age provided |
|  | Kadel (2024) | From Pixels to Palate: Communication around #vegan on Instagram and its relation with eating intentions. | Study design – doesn’t present data in a manner that can be extracted |
|  | Khan (2021) | Tea culture and industry: Customer tea buying decision-making power shaped by social capital in the presence of mutual trust. | Population – average, mean or media age not provided |
|  | Kinard (2016) | Insta-Grams: The Effect of Consumer Weight on Reactions to Healthy Food Posts. | Population – average age of sample = 32.1 |
|  | Kerrigan (2019) | 5-2-1-0 Lifestyle risk factors predict obesity in Millennials. | Exposure – lacks emphasis on digital food communication |
|  | Kuang-HengShih (2018) | Assessing the Relationship among Online Word-of-Mouth, Product Knowledge, and Purchase Intention in Chain Restaurant. | Population – participants aged 18-45+; no average, mean or median age provided |
|  | Krzyzanowski (2020) | Rams Have Heart, a mobile app tracking activity and fruit and vegetable consumption to support the cardiovascular health of college students: Development and usability study | Data Presentation – Unable to extract required data |
|  | Lang (2021) | Impact of an Innovative Video Demonstration on Perceptions and Attitudes Toward McDonald's Product Quality. | Population – participants were 15- 50+; mean, average or median not age provided; |
|  | Laska (2016) | A 2-year young adult obesity prevention trial in the US: Process evaluation results. | Population – wrong population |
|  | Liang (2022) | Psychological Mediators of Web-Based Interventions for Promoting a Healthy Lifestyle Among Chinese College Students: Secondary Analysis of a Randomised Controlled Trial | Study design – secondary analysis |
|  | Liu (2019) | Evaluating Mobile Health Apps for Customized Dietary Recording for Young Adults and Seniors: Randomized Controlled Trial. | Study design – focus on entry of food information vs responses to digital food communication |
|  | Long (2023) | Portion size affects food selection in an immersive virtual reality buffet and is related to measured intake in laboratory meals varying in portion size. | Population - mean age of participants is 29 |
|  | Lytle (2017) | Weight-gain reduction among 2-year college students: The CHOICES RCT. | Study design - Difference in delivery location of the course caused ambiguity if digital food communication caused the outcome |
|  | Martinez-Sala (2023) | Educommunication 2.0 in food and physical activity. Study of mobile applications in the fast food sector | Population – participants are people born from 1980s to 2000s. |
|  | Mayoh (2021) | Young people's experiences of engaging with fitspiration on Instagram: Gendered perspective. | Study design – Focuses on the difference in response to fitspiration content by gender |
|  | Meng (2017) | Online self-tracking groups to increase fruit and vegetable intake: A small-scale study on mechanisms of group effect on behavior change. | Study design – both intervention and control used digital food communication |
|  | Milan (2010) | Impact of a stage-tailored, Web-based intervention on folic acid-containing multivitamin use by college women. | Study design – intervention and control groups used digital food communication |
|  | Nelson (2019) | Gender differences in diet and social media: An explorative study | Missing data – does not present the number of participants randomised to each condition in the study |
|  | Nollen (2013) | Development and functionality of a handheld computer program to improve fruit and vegetable intake among low-income youth. | Population – participants aged 8 -15 |
|  | Nour (2019) | Young Adults' Engagement with a Self-Monitoring App for Vegetable Intake and the Impact of Social Media and Gamification: Feasibility Study. | Study design – intervention and control groups used digital food communication |
|  | Nour (2018) | Exploring young adult perspectives on the use of gamification and social media in a smartphone platform for improving vegetable intake. | Outcomes – lacks required behavioural/cognitive outcomes |
|  | Nguyen (2017) | Social media indicators of the food environment and state health outcomes. | Population – no details on age |
|  | O'Donnell (2014) | The Effect of Goal Setting on Fruit and Vegetable Consumption and Physical Activity Level in a Web-Based Intervention | Study design - utilises secondary data analysis |
|  | O'Kane (2022) | Investigating the feasibility and acceptability of using Instagram to engage post-graduate students in a mass communication social media-based health intervention, #WeeStepsToHealth. | Population – no mean age of participants |
|  | Pasko (2021) | The Roles of Social Comparison Orientation and Regulatory Focus in College Students' Responses to Fitspiration Posts on Social Media: Cross-sectional Study. | Study design – no focus on cognitive or behavioural responses to food or nutrition |
|  | Parks (2018) | Assessing the Feasibility of a Social Media to Promote Weight Management Engagement in Adolescents with Severe Obesity: Pilot Study | Population – participants aged 14-20; mean age = 16 |
|  | Piko (2022) | Risk of disordered eating in emerging adulthood: media, body and weight-related correlates among Hungarian female university students. | Exposure – lacks emphasis on digital food communication |
|  | Pollack (2021) | Twitch user perceptions, attitudes and behaviours in relation to food and beverage marketing on Twitch compared with YouTube | Population – No mean age of participants |
|  | Pope (2019) | Use of Wearable Technology and Social Media to Improve Physical Activity and Dietary Behaviors among College Students: A 12-Week Randomized Pilot Study. | Study design – intervention and control groups used digital food communication |
|  | Poppelaars (2018) | When winning is losing: A randomised controlled trial testing a video game to train food-specific inhibitory control | Data Presentation – unable to extract required data |
|  | Pryde (2022) | TikTok on the clock but the #fitspo don't stop: The impact of TikTok fitspiration videos on women's body image concerns. | Study design – lacks emphasis on food and nutrition |
|  | Raggatt (2018) | “I aspire to look and feel healthy like the posts convey”: engagement with fitness  inspiration on social media and perceptions of its influence on health and wellbeing | Study design – lacks emphasis on food and nutrition |
|  | Raja N (2022) | The relationship between the high-risk disordered eating and social network navigation among Saudi college females during the COVID pandemic. | Exposure – lacks emphasis on digital food communication |
|  | Rouf (2018) | Messaging for Interventions Aiming to Improve Calcium Intake in Young Adults-A Mixed Methods Study. | Study design – focus on how to target health messages instead of the impact of digital food communication |
|  | Rouf (2020) | Improving Calcium Knowledge and Intake in Young Adults Via Social Media and Text Messages: Randomized Controlled Trial. | Study design – intervention and control groups used digital food communication |
|  | Schwartz (2015) | Exploring the potential for internet-based interventions for treatment of overweight and obesity in college students | Study design – focus on places online where young people seek health information and trust of sources; not their cognitive or behavioural responses to this information |
|  | Sefa-Yeboah (2021) | Development of a Mobile Application Platform for Self-Management of Obesity Using Artificial Intelligence Techniques. | Population – no average, mean or media age provided |
|  | Stein (2021) | Every (Insta)Gram Counts? Applying Cultivation Theory to Explore the  Effects of Instagram on Young Users’ Body Image | Study design |
|  | Sullivan (2016) | A virtual reality intervention (Second Life) to improve weight maintenance: Rationale and design for an 18-month randomized trial. | Population – no average, mean or median provided |
|  | Supthanasup (2021) | Social networking sites: A new source of child feeding information for parents in Thailand. | Population – mean age = 35.0 |
|  | Tan (2016) | Internet and smartphone application usage in eating disorders: A descriptive study in Singapore. | Outcomes – lacks data showing the impact of digital food communication on health and behavioural responses |
|  | Tami (2022) | Perceived Effects of Socio-Economics and Social Media Variables on Body Mass Index in Saudi Young Adults. | Data Presentation – relevant data only presented as percentage responses to questions. |
|  | Tonkin (2017) | A Smartphone App to Reduce Sugar-Sweetened Beverage Consumption Among Young Adults in Australian Remote Indigenous Communities: Design, Formative Evaluation and User-Testing. | Population – no mean, average or median age provided |
|  | Tran (2023) | The impact of using food influencers on consumer purchase intention with attitude toward influencers as mediator. | Population - no mean, average or median age provided |
|  | Turner (2017) | Instagram use is linked to increased symptoms of orthorexia nervosa. | Study design – lacks emphasis on digital food communication |
|  | Villiard (2012) | Fitness on Facebook: Advertisements generated in response to profile content. | Study design – looks at ads generated based on participant profiles, not the impact on cognitive or behavioural responses |
|  | Vita (2020) | Do Millennials Believe in Food Vlogger Reviews? A Study of Food Vlogs as a Source of Information | Population – no average, mean or median age provided |
|  | Vita (2021) | The online marketing of Indonesia street food in Jakarta | Population – no average, mean or median age provided |
|  | Walker (2021) | A qualitative study exploring what it means to be healthy for young Indigenous Australians and the role of social media in influencing health behaviour. | Data presentation – qualitative quotes not presented on digital food communication to support study author conclusions |
|  | Wang (2021) | Promoting healthy lifestyle in Chinese college students: evaluation of a social media-based intervention applying the RE-AIM framework | Data presentation |
|  | Watanabe-Ito (2020) | Promoting Healthy Eating Habits for College Students Through Creating Dietary Diaries via a Smartphone App and Social Media Interaction: Online Survey Study | Study design – focus on awareness instead of impact on cognitive or behavioural responses |
|  | Werle (2024) | How a food scanner app influences healthy food choice. | Study design – use of the food scanner app was optional within the condition group |
|  | Wilson (2019) | Picture Yourself Healthy-How Users Select Mediated Images to Shape Health Intentions and Behaviors. | Study design – digital food communication is calculated as mediator of behaviour (independent variable) & intention (dependent variable), instead of digital food communication as the independent variable that impacts intention and behaviour as dependent variables |
|  | Worthington (2023) | Development of eHealth-based behavior change support for young adults using the Nine Principles framework. | Study design |
|  | Wu (2022) | An exploratory social media intervention for perception and behavior on eating away from home: A cluster randomized trial in Chongqing, China. | Outcome – relevant results table only provide pre/post values for the intervention and not control. It also only includes p values based on comparison with the intervention and control group which is insufficient information. |
|  | Xu (2021) | ‘I’m like you, and I like what you like’ sustainable food purchase influenced by vloggers: a moderated serial-mediation model. | Study design – the vlogger is the independent variable vs. the content |
|  | Zeeni (2021) | Social media use impacts body image and eating behavior in pregnant women. | Population – mean age = 29.6 |
|  | Zeeni (2024) | Exposure to instagram junk food content negatively impacts mood and cravings in young adults: A randomized controlled trial. | Missing data – lacks information on the number of participants in the control and intervention groups |
|  | Zorell (2022) | Central Persons in Sustainable (Food) Consumption. | Population – mean age = 17.92 |

## **Table 6**: Experimental Studies – Behavioural (Narratives of Outcome Data)

| STUDY ID | COUNTRY | STUDY DESIGN | POPULATION & MEAN AGE | SAMPLE SIZE | DIGITAL MEDIA | INTERVENTION | CONTROL | OUTCOME (S) | RESULTS | CONFIRMED HYPOTHESIS | POSITIVE SIGNIFICANT FINDINGS |
| --- | --- | --- | --- | --- | --- | --- | --- | --- | --- | --- | --- |
| Duan (2017) | China | Randomised trial | Undergrad. Students  [M =19] | 493 | Social media; website | Weekly web-based programme (8 weeks)  4 week – physical activity; 4 weeks – fruit & veg. intake | No  intervention | Behavioural – Food consumption  Tool: portions/day  Measurement: effect sizes and p values | Fruit and vegetable intake (portions/day)  Interaction time treatment  Effect size = .13  P value = <.001 | YES | YES |
| Duan (2022) | China | Randomised trial | College students  [M= 20] | Total = 565 | Social media; website | Intervention group 2 (FVC-first group: first 4-week intervention on FVC, followed by a 4-week intervention on physical activity; | Placebo control group | Behavioural – Food consumption  Tool: portions/day  Measurement: effect sizes and p values | FVC first vs control  P Value = < .001  Effect size = 0.59 | Yes | Yes |

## **Table 7**: Observational Studies – Behavioural (Narratives of Outcomes Data)

| STUDY ID | COUNTRY | STUDY DESIGN | POPULATION & MEAN AGE | SAMPLE SIZE | DIGITAL MEDIA | EXPOSURE | OUTCOME (S) | RESULTS | CONFIRMED HYPOTHESIS | POSITIVE SIGNIFICANT FINDINGS |
| --- | --- | --- | --- | --- | --- | --- | --- | --- | --- | --- |
| Chung (2021) | Hong Kong (China) | Controlled-experimental study | Young adults 18-39 in Hong Kong  [M = 22.1] | 305 | Social media; wearable device | Intervention: 3-hour nutrition seminar and 12 weeks dietary monitoring with mobile app  Control: 3-hour nutrition seminar only | Behavioural – Food consumption  Measurement: mean and standard deviation | Fruit consumption (grams)  Post intervention  Control = 26.1 (SD = 28.3)  Intervention = 61.6 (SD = 70.0) | Yes | Yes |
| Chung (2021) | Hong Kong (China) | Controlled-experimental study | Young adults 18-39 in Hong Kong  [M = 22.1] | 305 | Social media; wearable device | Intervention: 3-hour nutrition seminar and 12 weeks dietary monitoring with mobile app  Control: 3-hour nutrition seminar only | Behavioural – Food consumption  Measurement: mean and standard deviation | Vegetable consumption (grams)  Post intervention  Control = 26.1 (SD = 28.3)  Intervention = 456 (SD = 223) | Yes | Yes |
| Chung (2021) | Hong Kong (China) | Controlled-experimental study | Young adults 18-39 in Hong Kong  [M = 22.1] | 305 | Social media; wearable device | Intervention: 3-hour nutrition seminar and 12 weeks dietary monitoring with mobile app  Control: 3-hour nutrition seminar only | Behavioural – Food consumption  Measurement: mean and standard deviation | Dietary fibre intake (grams)  Post intervention  Control = 11.8 (SD = 4.78)  Intervention = 21.2 (SD = 11.56) | Yes | Yes |
| Chung (2021) | Hong Kong (China) | Controlled-experimental study | Young adults 18-39 in Hong Kong  [M = 22.1] | 305 | Social media; wearable device | Intervention: 3-hour nutrition seminar and 12 weeks dietary monitoring with mobile app  Control: 3-hour nutrition seminar only | Behavioural – Food consumption  Measurement: mean and standard deviation  Direction of effect: Control to be higher than intervention | Sugar intake (grams)  Post intervention  Control = 60.2 (SD = 34.4)  Intervention = 53.8 (SD = 36.9) | Yes | Yes |
| Chung (2021) | Hong Kong (China) | Controlled-experimental study | Young adults 18-39 in Hong Kong  [M = 22.1] | 305 | Social media; wearable device | Intervention: 3-hour nutrition seminar and 12 weeks dietary monitoring with mobile app  Control: 3-hour nutrition seminar only | Behavioural – Food consumption  Measurement: mean and standard deviation  Direction of effect: Control to be higher than intervention | Saturated fat intake (grams)  Post intervention  Control = 48.6 (SD = 17.5)  Intervention = 57.3 (SD = 29.4) | No |  |
| Coccia (2020) | U.S. A | Non  randomised  trial | Student athletes aged 18-24  [M = 22.1] | 27 | Social media; wearable device | 6-week nutritional education intervention on Twitter | Behavioural – Food consumption  Measurement: mean and standard deviation | Percentage of participant calories from fat at pre and post test  Pre= 31.36 (SD = 3.72)  Post = 30.07 (SD = 3.50)  P value = 0.032 | Yes | Yes |
| Coccia (2020) | U.S. A | Non  randomised  trial | Student athletes aged 18-24  (M = 22.1) | 27 | Social media; wearable device | 6-week nutritional education intervention on Twitter | Behavioural – Food consumption  Measurement: mean and standard deviation | Participant fruit and vegetable intake at pre and post test  Pre= 5.52 (SD = 3.00) Post = 6.80 (SD = 5.01) P value = 0.13 | Yes | No |
| Hawkins (2021) | United Kingdom | Experiment | Female college students  [M=20] | 169 | Social media | Exposure to a socially endorsed image using ‘likes’, consisting of: Low Energy Dense food images OR High Energy Dense food images.  Control images = interior design | Behavioural – Food consumption  Tool: Grams consumed  Measurement: mean and standard deviation | Grape consumption (grams)  Post-intervention  Control = 79.93 (SD = 58.79)  Intervention (Low Energy Dense) = 102.19 (SD = 81.76)  Calculated p-value = P = 0.1011 | Yes | No |
| Hawkins (2021) | United Kingdom | Experiment | Female college students  [M=20] | 169 | Social media | Exposure to a socially endorsed image using ‘likes’, consisting of: Low Energy Dense food images OR High Energy Dense food images.  Control images = interior design | Behavioural – Food consumption  Tool: Grams consumed  Measurement: mean and standard deviation | Cookie consumption (grams)  Post-intervention  Control = 21.6 (SD = 25.03)  Intervention (High Energy Dense) = 26.52 (SD = 26.23)  Calculated p-value: P = 0.1011 | Yes | No |
| Hilkens (2021) | Netherlands | Cross sectional | Male gym users  [M = 24] | 2269 | Social media | Social media and use of dietary supplements | Behavioural- Food consumption  Tool: Pearson Correlations  Measurement: standard error and p value | Impact of image-centric social media use on supplement use  B = 0.255  SE: 0.020  P = < 0.001 | Yes | Yes |
| Kilb (2023) | Germany | Randomised trial (non-randomised data) | 18–29 years  [M = 22] | 40 | Social media | Exposure to posts from study partner (sender). Control = B&M  Intervention = FVI | Behavioural- Food consumption  Tool: ANCOVA  Measurement: standard error and p value | Fruit and Vegetable Consumption  Control = -0.34 (SE =0.72)  Intervention = 1.00 (SE = 46) | Yes | No |
| Krishnamohan (2017) | India | Non  randomised  trial | Medical students aged 18-23 | 45 | Social media | Health education messages on Facebook (messages, pictures & videos) for six weeks. | Behavioural – Food consumption  Tool: adapted WHO STEPS Questionnaire  Measurement: mean and standard deviation | Post intervention  Intake of fruit [servings/day]  Control = 1.0 (SD = .6)  Intervention = 1.27 (SD = .7)  Calculated p-value: P = 0.1713 | Yes |  |
| Krishnamohan (2017) | India | Non  randomised  trial | Medical students aged 18-23 | 45 | Social media | Health education messages on Facebook (messages, pictures & videos) for six weeks. | Behavioural – Food consumption  Tool: adapted WHO STEPS Questionnaire  Measurement: mean and standard deviation | Post intervention  Intake of vegetables [servings/day]  Control = 1.2 (SD = .5)  Intervention = 1.1 (SD = .8)  Calculated p-value: P = 0.6160 | No |  |
| Krishnamohan (2017) | India | Non  randomised  trial | Medical students aged 18-23 | 45 | Social media | Health education messages on Facebook (messages, pictures & videos) for six weeks. | Behavioural – Food consumption  Tool: adapted WHO STEPS Questionnaire  Measurement: mean and standard deviation | Post intervention  Intake of junk food [times/day]  Control = 2.7 (SD = 2.5)  Intervention = 2 (SD = 2.5) | Yes |  |
| Pollack (2022) |  | Cross sectional | Over 13 and user of Twitch.tv  [Median = 21] | 568 | Social media – Twitch | Twitch | Behavioural – Food purchasing  Tool: External Food Cue Responsiveness Scale  [average of the nine scale values]  Measurement: Odds ratio and 95% Confidence Interval | Do you ever purchase products because you have seen them advertised on Twitch?  [External Food Cue Responsiveness Scale]  Odds Ratio = 3.97  95% CI = 1.99, 8.26  P value = 0.001 | Yes | Yes |
| Pollack (2022) |  | Cross sectional | Over 13 and user of Twitch.tv  [Median = 21] | 568 | Social media – Twitch | Twitch | Behavioural – Food purchasing  Tool: Dutch Eating Behaviour Questionnaire  [1 unit increase in scale]  Measurement: Odds ratio and 95% Confidence Interval | Do you ever purchase products because you have seen them advertised on Twitch?  [Dutch Eating Behaviour Questionnaire]  Odds Ratio = 1.05  95% CI = 0.94, 1.16  P value = 0.39 | Yes | No |
| Pollack (2022) |  | Cross sectional | Over 13 and user of Twitch.tv  [Median = 21] | 568 | Social media – Twitch | Twitch | Behavioural – Food purchasing  Tool: Three Factor Eating Questionnaire  [1 unit increase in scale]  Measurement: Odds ratio and 95% Confidence Interval | Do you ever purchase products because you have seen them advertised on Twitch?  [Three Factor Eating Questionnaire]  Odds Ratio = 1.01  95% CI = 0.99, 1.03  P value = 0.16 | Yes | No |
| Sharps (2019) | United Kingdom | Non randomised trial | University students in the United Kingdom  [M = 19.00] | 21 | Social media | 2 weeks exposure to images of peers’ high-energy-dense snacks and sugar-sweetened beverages on social media (Instagram) | Behavioural – food consumption  Tool: 6-point Likert-style scale from once per month or never to daily  Measurement: mean and standard deviation | Frequency of consumption of High Energy Dense Snacks  Baseline = 1.58 (SD = .33)  Post-Intervention = 1.51 (SD = .45)  Calculated p-value: P = 0.5686 | Yes | No |
| Sharps (2019) | United Kingdom | Non randomised trial | University students in the United Kingdom  [M = 19.00] | 21 | Social media | 2 weeks exposure to images of peers’ high-energy-dense snacks and sugar-sweetened beverages on social media (Instagram) | Behavioural – food consumption  Tool: 6-point Likert-style scale from once per month or never to daily  Measurement: mean and standard deviation | Frequency of consumption of Sugar Sweetened Beverages  Baseline = 2.12 (SD = .78)  Post-Intervention = 1.98 (SD = .81)  Calculated p-value: P = 0.5686 | Yes | No |
| Slazus (2022) | South Africa | Non  Randomised  trial | College students aged 18 - 25 | 61 | Mobile app | Use of MyFitnessPal for 3 weeks | Behavioural – food consumption  Tool: Adapted Dietary Instrument for Nutrition Education (DINE)  Measurement: Mean difference between before and after trial | Mean difference before and after use  Fatty Foods  Mean = -0.12  Standard Error = 1.09  95% C.I. = -2.30 to 2.05  Standard deviation= 8.48  P-value = 0.91 | No | No |
| Slazus (2022) | South Africa | Non  Randomised  trial | College students aged 18 - 25 | 61 | Mobile app | Use of MyFitnessPal for 3 weeks | Behavioural – food consumption  Tool: Adapted Dietary Instrument for Nutrition Education (DINE)  Measurement: Mean difference between before and after trial | Mean difference before and after use  Sugary Foods  Mean = 0.54  Standard Error = 0.24  Standard deviation= 1.84  P-value = 0.03 | Yes | Yes |
| Slazus (2022) | South Africa | Non  Randomised  trial | College students aged 18 - 25 | 61 | Mobile app | Use of MyFitnessPal for 3 weeks | Behavioural – food consumption  Tool: Adapted Dietary Instrument for Nutrition Education (DINE)  Measurement: Mean difference between before and after trial | Mean difference before and after use  Fruit and Vegetables  Mean = 0.01  Standard Error = 0.27  Standard deviation = 2.12  95% C.I. = -0.53 to 0.55  P-value = 0.98 | Yes | No |

## **Table 8:** Experimental Studies – Cognitive (Narratives of Outcomes Data)

| STUDY ID | COUNTRY | STUDY DESIGN | POPULATION & MEAN AGE | SAMPLE SIZE | DIGITAL MEDIA | INTERVENTION | CONTROL | OUTCOME (S) | RESULTS | CONFIRMED HYPOTHESIS | POSITIVE SIGNIFICANT FINDINGS^[[1]](#endnote-2)^ |
| --- | --- | --- | --- | --- | --- | --- | --- | --- | --- | --- | --- |
| Kinkel-Ram (2022) | U.S. A | Randomised trial | Female university students  [Site 1  M = 18:59]  Site 2  M = 23.70] | Site 1 = 222; Site 2 = 214 | Wearable device | Instagram feed featuring low calorie, aesthetically pleasing foods | Instagram feed featuring travel images | Cognitive – Intention to consume  Tool: Eating Disorder Behaviour Scale  Measurement: Mean square and p value | Eating disorder intentions  Site 1 = 435.97  P value = 0.39  Site 2 = 10.28  P value = 0.90 | No | No |

## **Table 9**: Observational Studies – Cognitive (Narratives of Outcomes Data)

| STUDY ID | COUNTRY | STUDY DESIGN | POPULATION & MEAN AGE | SAMPLE SIZE | DIGITAL MEDIA | EXPOSURE | OUTCOME (S) | RESULTS | CONFIRMED HYPOTHESIS | POSITIVE SIGNIFICANT FINDINGS |
| --- | --- | --- | --- | --- | --- | --- | --- | --- | --- | --- |
| Buchanan (2017) | Australia | Non  randomised trial | Young adults aged 18-24  [M = 20] | 60 | Social media; website | Intervention: websites + social media sites of two energy drink brands  Control: websites + social media sites of two nut bar brands | Cognitive – attitudes and perceptions towards food  Tool: Mean of five 7-point semantic-differential scales e.g.: “bad/good”,  Measurement: Mean and standard deviation | Attitude - energy drink products (general)  Experimental = 0.2 (SD = 1.5)  Control = -0.8 (SD = 1.7)  P = .028 | Yes | Yes |
| Buchanan (2017) | Australia | Non  randomised trial | Young adults aged 18-24  [M = 20] | 60 | Social media; website | Intervention: websites + social media sites of two energy drink brands  Control: websites + social media sites of two nut bar brands | Cognitive – Purchase intention  Tool: 5-point intention scale, ranging from “definitely will not purchase” to “definitely will purchase”  Measurement: Mean and standard deviation | Purchase intention - energy drink products (general)  Experimental = -1.0 (SD = 1.0)  Control = - 1.5 (SD = - 2.0)  P value = .108 | No | No |
| Buchanan (2018) | Australia | Cross sectional | People aged 18-24 | 359 | Social media; video games; website | Digital marketing of energy drinks | Cognitive – attitudes towards food  Tool: Theory of Planned Behaviour constructs  Measurement: Mediation analysis and p value | Attitude – energy drink products  Direct effect: β = .11  P value = < .05 | Yes | Yes |
| Buchanan (2018) | Australia | Cross sectional | People aged 18-24 | 359 | Social media; video games; website | Digital marketing of energy drinks | Cognitive – intention to consume  Tool: Theory of Planned Behaviour constructs  Measurement: mediation analysis and p value | Perceived behavioural control  Direct effect: β = –.13  P value = < .05 | Yes | Yes |
| Coccia (2020) | U.S. A | Non  randomised trial | Student athletes aged 18-24  [M = 22.1] | 28 | Social media; wearable device | 6-week nutritional education intervention on Twitter | Cognitive – intention to consume  Measurement: mean and standard deviation | Participant self-efficacy  Pre= 14.75 (3.41)  Post = 15.21 (3.96)  P value = 0.35 | Yes | No |
| Sharps (2019) | United Kingdom | Non randomised trial | University students in the United Kingdom | 21 | Social media | 2 weeks exposure to images of peers’ high-energy-dense snacks and sugar-sweetened beverages on social media (Instagram) | Cognitive – intention to consume  Tool: 5-point Likert-style scale from completely disagree to completely agree.  Measurement: mean and standard deviation | Participant intention to change consumption of High Energy Dense Snacks  Baseline = 3.53 (SD = 1.03)  Post-Intervention = 3.88 (SD = .92)  Calculated p-value: P = 0.2524 | Yes | No |
| Sharps (2019) | United Kingdom | Non randomised trial | University students in the United Kingdom | 21 | Social media | 2 weeks exposure to images of peers’ high-energy-dense snacks and sugar-sweetened beverages on social media (Instagram) | Cognitive – intention to consume  Tool: 5-point Likert-style scale from completely disagree to completely agree.  Measurement: mean and standard deviation | Intention to change consumption of Sugar Sweetened Beverages  Baseline = 2.80 (SD = 1.02)  Post-Intervention = 3.18 (SD = .98)  Calculated p-value: P = 0.2255 | Yes | No |
| Sharps (2019) | United Kingdom | Non randomised trial | University students in the United Kingdom | 21 | Social media | 2 weeks exposure to images of peers’ high-energy-dense snacks and sugar-sweetened beverages on social media (Instagram) | Cognitive – intention to consume  Tool: 5-point Likert-style scale from completely disagree to completely agree.  Measurement: mean and standard deviation | Participant desired portion size for High Energy Dense Snacks  Baseline = 1.47 (SD = .28)  Post-Intervention = 1.28 (SD = .27)  Calculated p-value: P = 0.0308 | Yes | Yes |
| Sharps (2019) | United Kingdom | Non randomised trial | University students in the United Kingdom | 21 | Social media | 2 weeks exposure to images of peers’ high-energy-dense snacks and sugar-sweetened beverages on social media (Instagram) | Cognitive – intention to consume  Tool: 5-point Likert-style scale from completely disagree to completely agree.  Measurement: mean and standard deviation | Intention to change consumption of High Energy Dense Snacks  Baseline = .88 (SD = .21)  Post-Intervention = .81 (SD = .27)  Calculated p-value: P = 0.2866 | Yes | No |

## **Table 10**: Behavioural Themes, Subthemes and Quotes

**RQ 1 - What is the impact of digital food communication on young people’s behavioural responses to food and nutrition?**

| THEMES | SUBTHEMES | QUOTATIONS/DATA |
| --- | --- | --- |
| IMPLEMENT TACTICS AND TOOLS TO EAT HEALTHY | Changed dietary behaviours | [<Files\\Vedovato et al. (2021)The “Comida de República” web-based>](file:///C:\Users\ShaniekP\AppData\Local\Microsoft\Windows\INetCache\Content.Outlook\BTPXMX2B\0a9907fa-0d1f-4e0e-addb-c32686e3eaa1) -  Reference 1  I started to better organize my eating time [...] and to choose a healthier meal, with better nutritional quality.  Reference 2  Choosing more natural foods like those at the open market, and trying to avoid industrialized foods. These were things that I already had in mind, and that the course reinforced, so that I wanted to put them into practice [...] more salads and fruits, taking my favorites to eat at university or at work.  [<Files\\Duan et al. (2022) The Effectiveness of Sequentially Delivered Web-Based Interventions on Promoting PA and FV Consumption Among Chinese College Students- Mixed Methods Study>](https://unioxfordnexus-my.sharepoint.com/personal/lina4229_ox_ac_uk/Documents/Systematic%20Review/a5572ffb-0a44-4e4f-89db-c326861bbff3)  Reference 1  In comparison, only one student (participant 18) in the control group reported an increase in this behavior: I eat more fruit and vegetables every day after participating in the health learning program...I pay more attention to this health issue now. [Participant 9]  [<Files\\Brennan (2020) A social marketing perspective of young adults concepts of eating for health - Is it a question of morality>](https://unioxfordnexus-my.sharepoint.com/personal/lina4229_ox_ac_uk/Documents/Systematic%20Review/b2d0f086-d812-46f3-84db-c32685a6f2e7) -  Reference 1  “Soon after I watched the documentary forks over knives and I was shocked into action! I researched a plant-based diet for weeks and never looked back. I started to see health as something that starts from the inside. How can you feed a body crap with a side of sugar and expect it to last for 80 years? I see health as 80% diet and 20% exercise. Now we eat an abundance of plants, grains, legumes, seeds and nuts and I have never felt healthier or stronger. I would like to implement more exercise. I have a 7 month old and any spare time is precious! I love talking about health and well-being. It’s exciting to me because I have gone from not really caring to be so passionate about it!” 24yo Female (F)  [<Files\\Nour et al. (2019) Young Adults Engagement with Self Monitoring App for vegetable Intake and the impact of social media and gamification. PDF>](https://unioxfordnexus-my.sharepoint.com/personal/lina4229_ox_ac_uk/Documents/Systematic%20Review/11768fc6-d4f5-4f1e-92db-c32686b5fd2c)  Reference 1  Most participants reported that the goal setting and self-monitoring features of the app were the most useful for increasing vegetable intake. As summarized by 1 male: I found it useful to set a goal of how many veggies to have a day and revise the target over time. |
|  | Changed relationships with food | [<Files\\Vedovato et al. (2021)The “Comida de República” web-based>](file:///C:\Users\ShaniekP\AppData\Local\Microsoft\Windows\INetCache\Content.Outlook\BTPXMX2B\0a9907fa-0d1f-4e0e-addb-c32686e3eaa1) -  Reference 1  I think the main change was how I started to see food [...] I reflected a lot on the relationship between human beings and food [...] and I started to eat more consciously.  Reference 2  Paying more attention to processed foods [...] decreasing the consumption of snacks and delivered ready-to-eat and fast foods [...] I stopped eating so many junky food, especially at the weekends [...] I stopped eating “noodles” and sausages [laughs] and I’m much more intolerant to industrialized products and “fast food”!  Reference 3  Observe the origin of the food, [...] paying more attention to the ingredients, quality and origin [...] concerned with looking at labels.  Reference 4  Giving more value to the act of cooking [...] and preparing more my own foods and dishes  [<Files\\Nour et al. (2019) Young Adults Engagement with Self Monitoring App for vegetable Intake and the impact of social media and gamification. PDF>](https://unioxfordnexus-my.sharepoint.com/personal/lina4229_ox_ac_uk/Documents/Systematic%20Review/11768fc6-d4f5-4f1e-92db-c32686b5fd2c)  Reference 1  Several participants expressed that they learnt to self-assess daily vegetable intake as well as the variety consumed within a couple of weeks of logging. They liked that the app gave them a benchmark goal to work against. A few mentioned that they now give consideration to what they eat throughout the day, and if their consumption of vegetables is low, they would compensate through the dinner meal. This is well summarized by 1 male who stated:  “I didn’t realize how many serves you are meant to eat and the variety, and now I think about the whole day, like I’ve had some this morning but none all day so I should have some at dinner.” |
|  | Utilising mobile apps to increase vegetable intake | [<Files\\Nour et al. (2019) Young Adults Engagement with Self Monitoring App for vegetable Intake and the impact of social media and gamification. PDF>](https://unioxfordnexus-my.sharepoint.com/personal/lina4229_ox_ac_uk/Documents/Systematic%20Review/11768fc6-d4f5-4f1e-92db-c32686b5fd2c) -  Reference 1  The young adults who were responsible for meal preparation indicated that the simple in-app recipes provided good ideas on how to cook with vegetables on a budget. As summarized by one female:  The recipes were so simple whereas recipes I look up online are not using my usual pantry items. I liked that I could use leftovers in the fridge especially since I am watching my budget a bit more. The pricing was good.  Reference 2  Most participants reported that the goal setting and self-monitoring features of the app were the most useful for increasing vegetable intake. As summarized by 1 male: I found it useful to set a goal of how many veggies to have a day and revise the target over time.  Reference 3  Despite the positive implications of self-monitoring, a majority of those interviewed also indicated that recording with the app discontinued toward the end of the 4-week period and this resulted in a slight drop in their consumption. As stated by 1 female:  It definitely helped to increase vegetable intake at the time when I was using the app, but since I stopped tracking I haven’t been accountable for my intake so I feel I am not as conscious.” It became apparent in the interviews that use of the app for self-monitoring is likely short-lived. Some stated that they would keep the app on their devices for access to recipes and meal ideas after the interventions end.  Reference 4  Logging helped me keep track of what I was eating and I did find sometimes the Facebook post would be a reminder to log (especially at the end of the day when I’m busy).” |
|  | Utilising tactics to limit exposure to unhealthy foods | [<Files\\Molenaar et al. (2021) Effects of Advertising - A Qualitative Analysis of Young Adults' Engagement with Social Media About Food>](https://unioxfordnexus-my.sharepoint.com/personal/lina4229_ox_ac_uk/Documents/Systematic%20Review/6f6e9599-edb4-4423-b9db-c32686965524)  Reference 1  The inability to consume a healthy diet, as the participants thought they should, often gave rise to feelings of guilt and shame as they mentioned they crave/could not resist “unhealthy” foods. “Most of the food ads I get are for fast food or delivery services, and I try to hide them when I see them so that I can avoid temptation!” (Forum 12: Female, 24 years old)  [<Files\\Vedovato et al. (2021)The “Comida de República” web-based>](file:///C:\Users\ShaniekP\AppData\Local\Microsoft\Windows\INetCache\Content.Outlook\BTPXMX2B\0a9907fa-0d1f-4e0e-addb-c32686e3eaa1) -  Reference 1  I started preparing lunchboxes at the weekend, so when I didn’t have access to the university restaurant, I didn’t choose junk food. |
| FOOD PURCHASES AND DIETARY BEHAVIOURS INFLUENCED BY ADVERTISEMENTS | Purchases influenced by advertisements | [<Files\\Friedman et al. (2022) The Use of Social Media as a Persuasive Platform to Facilitate Nutrition and Health Behavior Change in Young Adults - Web-Based Conversation Study>](file:///C:\Users\ShaniekP\AppData\Local\Microsoft\Windows\INetCache\Content.Outlook\BTPXMX2B\a7f0ee7d-ebb5-4b3c-abdb-c3268656ab6c)  Reference 1  Some participants who revealed the difficulties in resisting fast-food advertisements viewed their temptation as a lapse in self-discipline. A participant remarked the following: “I may have been ‘persuaded’ (read ‘reminded of my weak will’) to purchase [fast food brand name removed] on several occasions.” [persuasion forum; male; aged 18-21 years; low interest in health]  [<Files\\Vaterlaus et al. (2015) Getting Healthy - The percieved influence of social media on young adult health behaviours>](https://unioxfordnexus-my.sharepoint.com/personal/lina4229_ox_ac_uk/Documents/Systematic%20Review/aab09e5d-1410-45cb-91db-c32686d6fea2) -  Reference 1  A young adult (male, 21) observed how one of his friends was misled by a ‘‘get ripped quick’’ scheme. He stated:  [My friend] saw this ‘‘click here and get one free month of this supplement’’ [social media post] and he ordered it and he showed me. It was going to be absolutely terrible for him . . . He was pretty excited and I ended up throwing it away because it wasn’t healthy for him at all.  [<Files\\Molenaar et al. (2021) Effects of Advertising - A Qualitative Analysis of Young Adults' Engagement with Social Media About Food>](https://unioxfordnexus-my.sharepoint.com/personal/lina4229_ox_ac_uk/Documents/Systematic%20Review/6f6e9599-edb4-4423-b9db-c32686965524)  Reference 1  Some jingles and repetitive aspects of product promotion were also viewed as annoying, despite them being “catchy” and even, in some cases, inﬂuencing behaviour. “I guess that even if it’s annoying, the fact I remember it suggests that it worked. I even shop at [brand name removed], and recognise some of the specials.” (Forum 3: Male, 22 years old)  [<Files\\Leu et al.(2022)You know what, I'm in the trend as well-Understanding the interplay between digital & real-life social influences on the food & activity choices of YA>](https://unioxfordnexus-my.sharepoint.com/personal/lina4229_ox_ac_uk/Documents/Systematic%20Review/39ef073e-a1a8-49fd-94db-c32686754c65) -  Reference 1  [When] I’m looking for healthier choices. I actually use this quite a lot, the Healthier Choice sign. I think the government did a good job in implementing that. I actually look for that if I’m shopping for groceries, quite often, yeah.  – D01, male, 21, Chemical Engineering |
|  | Dietary behaviours influenced by advertisements | [<Files\\Molenaar et al. (2021) Effects of Advertising - A Qualitative Analysis of Young Adults' Engagement with Social Media About Food>](file:///C:\Users\ShaniekP\AppData\Local\Microsoft\Windows\INetCache\Content.Outlook\BTPXMX2B\6f6e9599-edb4-4423-b9db-c32686965524)  Reference 1  “I’ve seen the 2 fruit 5 veg ad on Facebook multiple times. Deﬁnitely something that I ﬁnd intriguing and occasionally try to do, but not always successfully.” (Forum 12: Male, 23 years old)  Reference 2  These young adults frequently described EDNP food products, with fewer participants recalling advertisements for healthy foods. Even when asked about health product advertising, participants often described unhealthy EDNP food or fast food instead. There was a sense of familiarity and afﬁnity towards a lot of the foods being described, with participants stating they already ate and enjoy these foods. Knowing how the food tastes sometimes meant the participants were more enticed by the advertisement. “I think it’s easier for food chains (especially the popular ones) to grab our attention because we already know what we’re getting, most of us would have tried their food before and know how delicious it is. So going back for a special deal or to try something new is motivating for us.” (Forum 3: Female, 22 years old)  [<Files\\Leu et al.(2022)You know what, I'm in the trend as well-Understanding the interplay between digital & real-life social influences on the food & activity choices of YA>](https://unioxfordnexus-my.sharepoint.com/personal/lina4229_ox_ac_uk/Documents/Systematic%20Review/39ef073e-a1a8-49fd-94db-c32686754c65) -  Reference 1  Especially McDonald’s, right, even if you don’t have it for a very long time. And then it release a new burger and your friend was like, “have you tried their new burger?” [participants laughed]. Well looks like I’m going there. Or they will jio [Singaporean slang for “invite”] you go. And, and it works, you know. It works so well. Uh, every time they do this promotional campaign, a bunch of people go just for the hype. [A few participants making “mm” sounds in agreement to what was said]: They [the marketing companies], they know it. They’re cashing in on it. There weren’t so many specialty burgers in the past. Now there’s like a few every year.  – D01, male, 21, Chemical Engineering |
|  | Purchases and dietary behaviours influenced by online promotions | [<Files\\Leu et al.(2022)You know what, I'm in the trend as well-Understanding the interplay between digital & real-life social influences on the food & activity choices of YA>](file:///C:\Users\ShaniekP\AppData\Local\Microsoft\Windows\INetCache\Content.Outlook\BTPXMX2B\39ef073e-a1a8-49fd-94db-c32686754c65) -  Reference 1  I don’t really look at these kinds of advertisements unless, unless it’s for promotions. Like, food promotions. Like, there was a period of time when there was, like, a lot, like, Uber Eats [an online meal ordering and delivery platform] discount codes, then they [the company] posted, then everyone just went, like you know, take advantage of the situation.  Reference 2  But then, um, I guess sometimes when you like scroll through Insta [Instagram] or what, then they have those adverts, you know those mini-adverts that they started doing. Then sometimes got food or like certain commercials on going out all that kind, yeah then I will, I would click and see ah. Yeah :: : sometimes ::: Like there was the :: : The Manhattan Fish Market was showing, and KFC also had the, you know those online coupon thingies :: : Yeah yeah, so they had that on Insta, so I was like, okay, I’ll get mine now.  – D05, male, 21, Economics |
| PEERS  INFLUENCE DIETARY BEHAVIOURS | Dietary behaviours influenced by peer posts and messages | [<Files\\Vaterlaus et al. (2015) Getting Healthy - The percieved influence of social media on young adult health behaviours>](https://unioxfordnexus-my.sharepoint.com/personal/lina4229_ox_ac_uk/Documents/Systematic%20Review/aab09e5d-1410-45cb-91db-c32686d6fea2) -  Reference 1  Seeing other peoples’ accomplishments in ‘‘#transformationtuesday’’ (i.e., posting before and after weight loss pictures) posts, pictures of progress through participation in speciﬁc exercise programs (e.g., Crossﬁt; see www.crossﬁt.com), and motivational quotes were also perceived to increase the personal desire to exercise. A young adult (male, 22) explained:  You see a little quote or something like ‘‘what are you doing with your life.’’ And you’re like hey I’m better than this I’m not going to bother eating this tub of ice cream. I’m going to go out there and run.    Reference 2  A young adult (male, 21) stated, ‘‘I actually use Twitter for good diets and eating habits so [Twitter] is beneﬁcial for me.’’ Participants indicated that there were good opportunities for healthy recipes on social media although they cautioned that the majority of recipes available were for ‘‘unhealthy foods,’’ ‘‘sweets,’’ and ‘‘desserts.’’ Social media was also credited with expanding food choices/recipes for speciﬁc diets like ‘‘paleo,’’ ‘‘vegan,’’ or for speciﬁc ‘‘food allergies.’’  [<Files\\Friedman et al. (2022) The Use of Social Media as a Persuasive Platform to Facilitate Nutrition and Health Behavior Change in Young Adults - Web-Based Conversation Study>](https://unioxfordnexus-my.sharepoint.com/personal/lina4229_ox_ac_uk/Documents/Systematic%20Review/a7f0ee7d-ebb5-4b3c-abdb-c3268656ab6c)  Reference 1  The young adults in our study noted that their peers influenced their health behaviors through both direct communication on the web and exposure to the content they posted on social media feeds. A participant shared the following: One of my friends would always message me to double check I was having breakfast and would always ask me what I had because she could tell when I was lying. [change forum; female; aged 18-21 years; low interest in health] |
|  | Showcasing health behaviours and food to peers through participation in online food culture | [<Files\\Vaterlaus et al. (2015) Getting Healthy - The percieved influence of social media on young adult health behaviours>](https://unioxfordnexus-my.sharepoint.com/personal/lina4229_ox_ac_uk/Documents/Systematic%20Review/aab09e5d-1410-45cb-91db-c32686d6fea2) -  Reference 1  Participants discussed that the practice of posting pictures of food on social media was to either entice others to want to make the food or to just showcase their own food preparation skills. A participant (male, 20) indicated that on Instagram, ‘‘the big hashtag they use is #foodporn. They want you to look at it, and be like, ‘That looks delicious!’’’ Illustrating the other major reason for posting food a young adult (female, 21) disclosed, ‘‘I don’t like to cook, so it’s like ‘Oh, I outdid myself’ so I’m actually going to share it because it actually looks edible.’’  [<Files\\Leu et al.(2022)You know what, I'm in the trend as well-Understanding the interplay between digital & real-life social influences on the food & activity choices of YA>](https://unioxfordnexus-my.sharepoint.com/personal/lina4229_ox_ac_uk/Documents/Systematic%20Review/39ef073e-a1a8-49fd-94db-c32686754c65) -  Reference 1  I think, sometimes when I see those café stuff, right, I feel like people don’t always go there just to try them, but out of social pressure. It’s like everybody’s going there, so it’s like I have to go there and try also, otherwise I would be left out. Then people will be talking about it like Art Box [A huge flea market with more than 300 fashion and food stalls that happened from April 14th – 23rd, 2017]. Like, initially I don’t think a lot of people was interested in it. Most people go out of social pressure because all their friends are posting on Instagram, like “I am going to Art Box, trying new foods”, so like, okay, you know what, I should go and try also. Even though people say it sucks, but everybody is going, so I just go :: : Yeah, it’s like to show people that “you know what, I’m in the trend as well”. |

## **Table 11**: Cognitive Themes, Subthemes and Quotes

**RQ 2 - What is the impact of digital food communication on young people’s COGNITIVE responses to food and nutrition?**

| THEMES | SUBTHEMES | QUOTATIONS/DATA |
| --- | --- | --- |
| FOOD CHOICES INFLUENCED BY COST | Cost viewed as a barrier to a healthy eating | [<Files\\Easton et al. (2018) - Young People's Experience of Viewing the Fitspiration SM Trend - Qualitative Study>](https://unioxfordnexus-my.sharepoint.com/personal/lina4229_ox_ac_uk/Documents/Systematic%20Review/bc617050-8526-44c3-8edb-c32686333bb0)  Reference 1  Several participants (whether students or employed) considered cost as a barrier to living a healthy lifestyle, especially because of the perceived cost of the food, gym memberships, and clothing.  I think something that’s not taken into account is that being fit is like money as well, like gym membership and gym clothes and healthy food, that’s so much money so if you can’t afford it you feel like, well I feel like there’s no point. [P9, F, 18]  [<Files\\Molenaar et al. (2021) Effects of Advertising - A Qualitative Analysis of Young Adults' Engagement with Social Media About Food>](https://unioxfordnexus-my.sharepoint.com/personal/lina4229_ox_ac_uk/Documents/Systematic%20Review/6f6e9599-edb4-4423-b9db-c32686965524)  Reference 1  Participants often reported noticing advertisements that highlighted affordable deals, discounts, and special offers. These participants often described themselves as students or on a low income and therefore the affordability of food and budgeting were major factors in their purchasing behaviours.  “On a budget as a university student the only ads that catch my attention are either cheap or on a good deal, like ads about [fast food brand name removed], [fast food brand name removed], [fast food brand name removed] etc. I am not particularly happy with my food choices, but eating healthy can’t be achieved by comparatively expensive healthy options to unhealthy ones.” (Forum 3: Male, 19 years old) |
|  | Budget- friendly fast food advertisements influence purchase intention | [<Files\\Molenaar et al. (2021) Effects of Advertising - A Qualitative Analysis of Young Adults' Engagement with Social Media About Food>](https://unioxfordnexus-my.sharepoint.com/personal/lina4229_ox_ac_uk/Documents/Systematic%20Review/6f6e9599-edb4-4423-b9db-c32686965524)  Reference 1  Participants stated that advertisements that promoted deals were sometimes viewed as deceptive rather than the good value for money that was intended. Some participants were sceptical that special deals actually saved money and believed it was more likely to lure them in but then they would spend more than intended.  “Creating ‘meal deals’ is one of the best things fast food places have done. It makes people feel as though they are getting a good deal, while walking away having spent more money and eating more calories than they initially planned.” (Forum 3: Female, 24 years old)  Reference 2  While value advertising strategies were appealing, and initially sounded like a cost- saving purchase, participants described how they usually end up buying more. This was especially mentioned in relation to fast-food companies with “meal deal”-type promotions. “The marketing they are using is to get people to visit the restaurants, people like myself are unlikely to spend only $3 because we consider this a good deal, it is likely that the more gullible of us (like myself) will spend more money than intended at the restaurant.” (Forum 3: Female, 24 years old)  Reference 3  Value for money often caught the eye of the participants as it was relevant to their needs. Price tempted participants to want whatever the advertisement was selling, and some participants stated price was the ultimate reason for them purchasing the advertised food. Even when speciﬁcally asked to recall advertisements about health, discounts and value for money of fast food and EDNP food were mentioned.  “With all the promos and deals that most junk food restaurants offer, they make it seems like a big bargain which sometimes it is, so it makes you want to go out and buy it.” (Forum 3: Male, 21 years old)  [<Files\\Friedman et al. (2022) The Use of Social Media as a Persuasive Platform to Facilitate Nutrition and Health Behavior Change in Young Adults - Web-Based Conversation Study>](https://unioxfordnexus-my.sharepoint.com/personal/lina4229_ox_ac_uk/Documents/Systematic%20Review/a7f0ee7d-ebb5-4b3c-abdb-c3268656ab6c)  Reference 1  These [fast-food] meals are cheap and easy, [and] although they’re [sic] aren’t healthy I know they will taste good. This [fast-food] advertising is very persuasive as it makes me believe that I am hungry and I cannot [sic] stop thinking about the new promotion. [persuasion forum; male; aged 18-21 years; moderate and high interest in health] |
| HEALTHY EATING INFORMATION IMPACTS AWARENESS & MOTIVATION | Exposure to healthy eating information influences awareness & motivation | [<Files\\Easton et al. (2018) - Young People's Experience of Viewing the Fitspiration SM Trend - Qualitative Study>](https://unioxfordnexus-my.sharepoint.com/personal/lina4229_ox_ac_uk/Documents/Systematic%20Review/bc617050-8526-44c3-8edb-c32686333bb0)  Reference 1  I’m a lot more aware of food groups, the whole ideal food groups plate arrangement, it’s like half vegetables, a quarter of protein, a quarter of carbs, I’m very aware of doing that when I have my dinners. [P6, F, 20] I think it has made me a lot more wary of what I put into my body but then I will have blow out days and just like literally shove food down. [P5, F, 20]  [<Files\\Friedman et al. (2022) The Use of Social Media as a Persuasive Platform to Facilitate Nutrition and Health Behavior Change in Young Adults - Web-Based Conversation Study>](https://unioxfordnexus-my.sharepoint.com/personal/lina4229_ox_ac_uk/Documents/Systematic%20Review/a7f0ee7d-ebb5-4b3c-abdb-c3268656ab6c)  Reference 1  I see a lot about healthy lifestyle and fitness in my social media feeds and I think that constant exposure has made me much more conscious of the choice I make, and a bit more aware of exercising and eating healthy. [persuasion forum; female; aged 22-24 years; low interest in health]  [<Files\\Duan et al. (2022) The Effectiveness of Sequentially Delivered Web-Based Interventions on Promoting PA and FV Consumption Among Chinese College Students- Mixed Methods Study>](https://unioxfordnexus-my.sharepoint.com/personal/lina4229_ox_ac_uk/Documents/Systematic%20Review/a5572ffb-0a44-4e4f-89db-c326861bbff3) -  Reference 1  In total, 9 of the 18 (50%) students recognized that consuming enough fruit and vegetables could help, while 6 students indicated the positive influence of regular PA on improving their perceived quality of life (Multimedia Appendix 8). For instance:  After participating in the health learning program, I exercised more, it brought me a good spiritual outlook...I felt that I slept better...Greasy food made me uncomfortable and fresh fruit and vegetables improved my well-being. [Participant 2]  [<Files\\Vaterlaus et al. (2015) Getting Healthy - The percieved influence of social media on young adult health behaviours>](https://unioxfordnexus-my.sharepoint.com/personal/lina4229_ox_ac_uk/Documents/Systematic%20Review/aab09e5d-1410-45cb-91db-c32686d6fea2)  Reference 1  Participants indicated that recipes are readily available on Pinterest, Facebook, and Twitter. A young adult (female, 21) revealed that social media: ... gives you more ideas to work with. I mean, let’s be honest, we pretty much all get into our ‘eating habits’ and they may not always be the best or they may be really boring. We eat the same stuff so sometimes it’s like, ‘‘that sounds really good I’m going to try it.’’ [Social media] may open this realm of taste that you never have experienced.  [<Files\\Nour et al. (2019) Young Adults Engagement with Self Monitoring App for vegetable Intake and the impact of social media and gamification. PDF>](https://unioxfordnexus-my.sharepoint.com/personal/lina4229_ox_ac_uk/Documents/Systematic%20Review/11768fc6-d4f5-4f1e-92db-c32686b5fd2c)  Reference 1  Recipes and tips posted on the Facebook group helped but the app was the most motivating to help me achieve my goals and seeing my progress.  Reference 2  Several participants expressed that they learnt to self-assess daily vegetable intake as well as the variety consumed within a couple of weeks of logging. They liked that the app gave them a benchmark goal to work against. A few mentioned that they now give consideration to what they eat throughout the day, and if their consumption of vegetables is low, they would compensate through the dinner meal. This is well summarized by 1 male who stated:  “I didn’t realize how many serves you are meant to eat and the variety, and now I think about the whole day, like I’ve had some this morning but none all day so I should have some at dinner.”  [<Files\\Vedovato et al. (2021)The “Comida de República” web-based>](https://unioxfordnexus-my.sharepoint.com/personal/lina4229_ox_ac_uk/Documents/Systematic%20Review/0a9907fa-0d1f-4e0e-addb-c32686e3eaa1)  Reference 1  I lack determination to reduce the junk food. [laughter]. |
|  | Exposure to online role models and information motivates healthy eating | [<Files\\Easton et al. (2018) - Young People's Experience of Viewing the Fitspiration SM Trend - Qualitative Study>](https://unioxfordnexus-my.sharepoint.com/personal/lina4229_ox_ac_uk/Documents/Systematic%20Review/bc617050-8526-44c3-8edb-c32686333bb0)  Reference 1  Participants described how Fitspiration content boosted their motivation to attend a gym, follow a nutritious diet, and helped them to adopt a positive mind-set. They described how motivation could be explicitly triggered by written, inspirational quotes. It helps me to set targets [...] see what I need to be doing and then kind of get me the road to doing it. [P3, F, 19]  In addition, observing posters helped them attain their goals and boosted motivation for working toward their health targets, and individuals posting Fitspiration content acted as aspirational figures and role models. I think they can be good for getting you motivated like definitely, […], if I try hard I could look like this. [P5, F, 20]  [<Files\\Friedman et al. (2022) The Use of Social Media as a Persuasive Platform to Facilitate Nutrition and Health Behavior Change in Young Adults - Web-Based Conversation Study>](https://unioxfordnexus-my.sharepoint.com/personal/lina4229_ox_ac_uk/Documents/Systematic%20Review/a7f0ee7d-ebb5-4b3c-abdb-c3268656ab6c)  Reference 1  The participants also described willpower as an important moderator of the influence that social media content had on their behavior. A participant believed the following:  If I try hard enough to work on my eating and doing more exercise then I will be able to be like them [models] with their hundreds of likes on their photos. [persuasion forum; male; aged 18-21 years; moderate and high interest in health]  Reference 2  Web-based health communities could motivate these highly driven participants to remain self-disciplined and self-reliant, which helped them to resist negative external influences such as fast-food advertisements. A participant explained the following:  I find it’s easier to stay motivated if I stay home and in routine (without access to bad food of course), and interact regularly with the online fitness/health community. [change forum; female; aged 22-24 years; moderate and high interest in health]  [<Files\\Leu et al.(2022)You know what, I'm in the trend as well-Understanding the interplay between digital & real-life social influences on the food & activity choices of YA>](https://unioxfordnexus-my.sharepoint.com/personal/lina4229_ox_ac_uk/Documents/Systematic%20Review/39ef073e-a1a8-49fd-94db-c32686754c65)  Reference 1  Yeah, and they also have, like, uh trainer, they will like promote themselves on Instagram, like they will suddenly pop up at the explorer thingy. Yeah, so like, I follow them ah, like :: : It motivates me to like, exercise and eat healthy. And like, when you see your friends also, like they, their body, or like nice right? Yeah, then like, make me feel down also lah, sometimes. But it also boost you up ah actually. Why you laugh? [participants laughed] Yeah, it actually motivate you ah, to like, have that body you know. Like yeah. – F04, female, 19, Infocomm Security Management |
|  | Recipes inspire meal considerations | [<Files\\Vaterlaus et al. (2015) Getting Healthy - The percieved influence of social media on young adult health behaviours>](https://unioxfordnexus-my.sharepoint.com/personal/lina4229_ox_ac_uk/Documents/Systematic%20Review/aab09e5d-1410-45cb-91db-c32686d6fea2)  Reference 1  Participants indicated that recipes are readily available on Pinterest, Facebook, and Twitter. A young adult (female, 21) revealed that social media: ... gives you more ideas to work with. I mean, let’s be honest, we pretty much all get into our ‘eating habits’ and they may not always be the best or they may be really boring. We eat the same stuff so sometimes it’s like, ‘‘that sounds really good I’m going to try it.’’ [Social media] may open this realm of taste that you never have experienced.  [<Files\\Nour et al. (2019) Young Adults Engagement with Self Monitoring App for vegetable Intake and the impact of social media and gamification. PDF>](https://unioxfordnexus-my.sharepoint.com/personal/lina4229_ox_ac_uk/Documents/Systematic%20Review/11768fc6-d4f5-4f1e-92db-c32686b5fd2c)  Reference 1  Recipes and tips posted on the Facebook group helped but the app was the most motivating to help me achieve my goals and seeing my progress.  [<Files\\Malloy et al. (2024) Empowering Young Women: A Qualitative Co-Design Study of a Social Media Health Promotion Programme. PDF>](https://unioxfordnexus-my.sharepoint.com/personal/lina4229_ox_ac_uk/Documents/Systematic%20Review/11768fc6-d4f5-4f1e-92db-c32686b5fd2c)  “It is quite refreshing to see videos with quite normal ingredients. Quite often, when I get these videos on my social media, it looks amazing but it’s really expensive ingredients and takes a lot of time.”—young woman 11 |
| DIGITAL INTERVENTIONS POSITIVELY IMPACT EATING CONSIDERATIONS | Exposure to virtual foods can change attitudes towards disordered eating | [<Files\\Cardi et al. (2012) - The Use of a Nonimmersive Virtual Reality Programme in Anorexia Nervosa A>](https://unioxfordnexus-my.sharepoint.com/personal/lina4229_ox_ac_uk/Documents/Systematic%20Review/218fdbd9-649e-48ed-97db-c32685fab344)  Reference 1  I remember when I first sat there with Isabel and Valentina my facial expression said it all, I found the food disgusting and never imagined being able to virtually eat the chocolate cake and pizza, let alone in real life. Having said this, the longer I sat there, the more it calmed my nerves about high content foods being in the fridge with lower calorie salads etc...That day I ate the salad and I think so the fish or fruit from the fridge, I had not reservations about this as I’d been living on that for a while now and for some reason the idea of it being in a fridge with chocolate cake extra did not faze me too much. That weekend I diminished my own fridge at home and me and mum went shopping with the same basket to buy are foods – an immediate difference noticed by my mum.  Reference 2  The virtual food was a gentle way of easing me back into being around high calorie and high content foods. The more I was around these foods, the more I became accustomed to seeing them in everyday life – it has become more normal to me nowadays and this definitely helped me with going to supermarkets and just being in kitchens with normal food around. I guess the understanding that these foods will always be around is now more of a comfort to me. I try to remember that it’s not imperative to finish a meal if your full and this keeps me more grounded, so I don’t over-indulge  Reference 3  I have tried various maudsley methods at overcoming my demons; however, stand by the fact that I believe the virtual fridge was of the most help. It got me back in touch with food again and made me more at ease with all foods and being around different types of healthy and non healthy indulgences |
|  | Participation in digital intervention improves wellbeing | [<Files\\Duan et al. (2022) The Effectiveness of Sequentially Delivered Web-Based Interventions on Promoting PA and FV Consumption Among Chinese College Students- Mixed Methods Study>](https://unioxfordnexus-my.sharepoint.com/personal/lina4229_ox_ac_uk/Documents/Systematic%20Review/a5572ffb-0a44-4e4f-89db-c326861bbff3) -  Reference 1  In total, 9 of the 18 (50%) students recognized that consuming enough fruit and vegetables could help, while 6 students indicated the positive influence of regular PA on improving their perceived quality of life (Multimedia Appendix 8). For instance:  After participating in the health learning program, I exercised more, it brought me a good spiritual outlook...I felt that I slept better...Greasy food made me uncomfortable and fresh fruit and vegetables improved my well-being. [Participant 2] |
| peer messages influence dietary CONSIDERATIONS | Peers influence dietary intentions | [<Files\\Leu et al.(2022)You know what, I'm in the trend as well-Understanding the interplay between digital & real-life social influences on the food & activity choices of YA>](https://unioxfordnexus-my.sharepoint.com/personal/lina4229_ox_ac_uk/Documents/Systematic%20Review/39ef073e-a1a8-49fd-94db-c32686754c65) -  Reference 1  While in some instances, posts by friends initiated or reinforced health-promoting behaviours, a more commonly shared experience was being tempted by shared posts with new and promotional food deals as experienced by F06 (female, 18, aviation management), ‘Like yeah, you get tempted by your friends like Instagram post like, they’ll post like, uh, “try this place”, “it’s a new place”, then they post the food, then like, you [are]also like tempted to go and buy’.  [<Files\\Friedman et al. (2022) The Use of Social Media as a Persuasive Platform to Facilitate Nutrition and Health Behavior Change in Young Adults - Web-Based Conversation Study>](https://unioxfordnexus-my.sharepoint.com/personal/lina4229_ox_ac_uk/Documents/Systematic%20Review/a7f0ee7d-ebb5-4b3c-abdb-c3268656ab6c)  Reference 1  Many participants shared that peer influence on social media drove both positive and negative health behaviors based on the content being shared:  Knowing what my friends eat...can encourage me to eat certain things. When a person shares an exercise [post], I am more inclined myself to exercise...when a friend makes a comment on a [Fast food brand name removed]’s post, I am more inclined to check out their deals. [persuasion forum; male; aged 22-24 years; moderate and high interest in health] |
|  | Peer feedback modulates effect of online content | [<Files\\Leu et al.(2022)You know what, I'm in the trend as well-Understanding the interplay between digital & real-life social influences on the food & activity choices of YA>](https://unioxfordnexus-my.sharepoint.com/personal/lina4229_ox_ac_uk/Documents/Systematic%20Review/39ef073e-a1a8-49fd-94db-c32686754c65)  Reference 1  Um, for me, I rarely notice um apps related to fitness, but maybe food, but usually I don’t really respond to these apps unless, um, there’s promotion, or I’m interested in it, or my friends have talked about it, yeah.  – C07, female, 23, Economics  Reference 2  I’m not sure if y’all know [popular local lifestyle blogger]. Like when the Chizza [Chizza is a portmanteau of ‘chicken’ and ‘pizza’. This product was sold at Kentucky Fried Chicken in Singapore] just came out and she was eating it, I wanted to try it. Yeah. So that was one of the factors of advertisements that was quite good I think :: : Lucky I didn’t [try] ’cause my friend told me it’s not nice.  – A02, female, 23, Project and Facilities Management |
|  | Reassurance derived from social media ‘commenters’/virtual peers | [<Files\\Brennan (2020) A social marketing perspective of young adults concepts of eating for health - Is it a question of morality>](https://unioxfordnexus-my.sharepoint.com/personal/lina4229_ox_ac_uk/Documents/Systematic%20Review/b2d0f086-d812-46f3-84db-c32685a6f2e7)  Reference 1  There were also those who were looking for signs of reassurance from others that they could be ‘good’ too. For example,  “I like seeing real people who comment and post to show that what they’ve been told is in fact working! I found it really interesting though, how many people also struggling with maintaining healthy meal schedules too! I for one, will either not eat at all through the day from being too busy, and then will splurge at night, or I’ll eat all the wrong foods!” 18yo F |
| Perceptions of FOOD-Related MARKETING & Messages | Marketing tactics influence perceptions | [<Files\\Buchanan et al. (2017) Exposure to digital marketing enhances young adults' interest in energy drinks - An exploratory investigation>](https://unioxfordnexus-my.sharepoint.com/personal/lina4229_ox_ac_uk/Documents/Systematic%20Review/e3cdf17c-185d-4bf8-92db-c32685e698da)  Reference 1  The superhero theme of V Energy appeared to be a selling point for some participants. “I am into Avengers (movie) that would convince me to purchase V [VEnergy] over Red Bull. . .” (participant d, female, 18 years).  Reference 2  The majority of participants favoured the “honesty” of the brands, for example, “V [V Energy] showed their nutrient contents, I was surprised by the low guarana content, it is not shady at all, really appealing”. (participant b, male, 18 years)  [<Files\\Molenaar et al. (2021) Effects of Advertising - A Qualitative Analysis of Young Adults' Engagement with Social Media About Food>](https://unioxfordnexus-my.sharepoint.com/personal/lina4229_ox_ac_uk/Documents/Systematic%20Review/6f6e9599-edb4-4423-b9db-c32686965524)  Reference 1  There were certain aspects of the food promotion that appeared to commonly grab and maintain the attention of these young adults. Frequency and repetition of an advertisement was effective in gaining the attention and sticking in the memory of these young adults. “As many have already said, the [fast food brand name removed] ad comes to my mind ﬁrst. The new [fast food product name removed] ad... has come on the many times that my mouth waters and I get the urge to go past [fast food brand name removed] hahaha.” (Forum 3: Female, 20 years old)  Reference 2  A combination of high frequency and foods that were relevant and appealing to them was described as a catalyst for purchasing a particular food. Promotions that were visually appealing, made the food look delicious, and used “happy” or bright colours were mentioned commonly.  “I think this was down to the colours. The ad used pastel colours which made it very easy on the eyes so it was eye catching and pleasant to look at—I wanted to stop and see what the ad was for” (Forum 3: Female, 21 years old)  [<Files\\Friedman et al. (2022) The Use of Social Media as a Persuasive Platform to Facilitate Nutrition and Health Behavior Change in Young Adults - Web-Based Conversation Study>](https://unioxfordnexus-my.sharepoint.com/personal/lina4229_ox_ac_uk/Documents/Systematic%20Review/a7f0ee7d-ebb5-4b3c-abdb-c3268656ab6c)  Reference 1  These [fast-food] meals are cheap and easy, [and] although they’re [sic] aren’t healthy I know they will taste good. This [fast-food] advertising is very persuasive as it makes me believe that I am hungry and I cannot [sic] stop thinking about the new promotion. [persuasion forum; male; aged 18-21 years; moderate and high interest in health]  [<Files\\Lambert et al. (2019) In their own words: A qualitative study exploring influences on the food choices of university students>](https://unioxfordnexus-my.sharepoint.com/personal/lina4229_ox_ac_uk/Documents/Systematic%20Review/a7f0ee7d-ebb5-4b3c-abdb-c3268656ab6c)  Reference 1  Yes. It's about appearance more than anything else…in advertising they use very slim attractive people, and you think, “Oh if I want to look like that then I should eat this” or “I shouldn't eat this”… [FG1] |
|  | Fast food ads perceived as negative and annoying | [<Files\\Friedman et al. (2022) The Use of Social Media as a Persuasive Platform to Facilitate Nutrition and Health Behavior Change in Young Adults - Web-Based Conversation Study>](https://unioxfordnexus-my.sharepoint.com/personal/lina4229_ox_ac_uk/Documents/Systematic%20Review/a7f0ee7d-ebb5-4b3c-abdb-c3268656ab6c)  Reference 1  Most ads on Facebook influence my health negatively...as they are usually for unhealthy food options. [persuasion forum; male; aged 22-24 years; low interest in health]  [<Files\\Molenaar et al. (2021) Effects of Advertising - A Qualitative Analysis of Young Adults' Engagement with Social Media About Food>](https://unioxfordnexus-my.sharepoint.com/personal/lina4229_ox_ac_uk/Documents/Systematic%20Review/6f6e9599-edb4-4423-b9db-c32686965524)  Reference 1  “I have recently seen many ads on [fast food brand name removed] mainly on Facebook, I ﬁnd it very annoying I also see it a lot on tv I never really see healthy food ads on tv or in any media I use daily.” (Forum 3: Female, 22 years old)  Reference 2  High frequency promotion was viewed as “annoying”, sometimes even considered “aggressive”, and generally decreased the likelihood that they would go and purchase the advertised food. “It played sometimes 3 times an ad break. Once I saw it back-to-back twice. It was exhausting.” (Forum 3: Female, 24 years old) |
|  | Exposure to fast food advertisements disrupt intentions for healthy eating | [<Files\\Molenaar et al. (2021) Effects of Advertising - A Qualitative Analysis of Young Adults' Engagement with Social Media About Food>](https://unioxfordnexus-my.sharepoint.com/personal/lina4229_ox_ac_uk/Documents/Systematic%20Review/6f6e9599-edb4-4423-b9db-c32686965524)  Reference 1  Participants often discussed the perceived healthfulness of the foods being advertised to them. Some participants communicated their discontent with being constantly exposed to fast-food advertisements, as these advertisements also hindered their ability to make healthy choices. “I’m trying to eat healthier and all I see is fast food around me. That really makes it difﬁcult to stay motivated and to avoid derailing back into unhealthy takeaway option.” (Forum 3: Female, 18 years old)  [<Files\\Friedman et al. (2022) The Use of Social Media as a Persuasive Platform to Facilitate Nutrition and Health Behavior Change in Young Adults - Web-Based Conversation Study>](https://unioxfordnexus-my.sharepoint.com/personal/lina4229_ox_ac_uk/Documents/Systematic%20Review/a7f0ee7d-ebb5-4b3c-abdb-c3268656ab6c) -  Reference 1  In relation to health and lifestyle it [social media] has not at all helped because it always shows videos of tasty unhealthy recipes and ads for [Fast food brand name removed] and [Fast food brand name removed]...It also then shows me photos of tall, tan, skinny models which makes me feel so bad about eating all the fast food. [persuasion forum; female; aged 18-21 years; low interest in health] |
|  | Dieting-related messages impacts body perceptions | [<Files\\Lambert et al. (2019) In their own words: A qualitative study exploring influences on the food choices of university students>](https://unioxfordnexus-my.sharepoint.com/personal/lina4229_ox_ac_uk/Documents/Systematic%20Review/a7f0ee7d-ebb5-4b3c-abdb-c3268656ab6c)  Reference 1  Both males and females commented that the majority of nutrition information on social media was accompanied by images of thin females and muscular males “looking fit, healthy and happy.” These images were associated with participants’ knowledge about what contributed to being healthy:  Slim fit girls are presented on social media as being in their active wear, drinking a smoothie. Fit muscular guys are all about the supplements, how to get large. [MG4]  Reference 2  Yes. It's about appearance more than anything else…in advertising they use very slim attractive people, and you think, “Oh if I want to look like that then I should eat this” or “I shouldn't eat this”… [FG1]  Reference 3  Perfect bodies on the paleo diet. Stuff like that is all over social media. [FG4]  [<Files\\Molenaar et al. (2020) Language of Health of Young Australian Adults: A Qualitative Exploration of Perceptions of Health,](https://unioxfordnexus-my.sharepoint.com/personal/lina4229_ox_ac_uk/Documents/Systematic%20Review/a7f0ee7d-ebb5-4b3c-abdb-c3268656ab6c)  [Wellbeing and Health Promotion via Online Conversations>](https://unioxfordnexus-my.sharepoint.com/personal/lina4229_ox_ac_uk/Documents/Systematic%20Review/a7f0ee7d-ebb5-4b3c-abdb-c3268656ab6c)  Alot of well being content is about looking “healthy” to impress people on Instagram, rather than what is actually good for you or realistic.” (Forum 4: Female, 24 years old).    [<Files\\Malloy et al. (2024) Empowering Young Women: A Qualitative Co-Design Study of a Social Media Health Promotion Programme. PDF>](https://unioxfordnexus-my.sharepoint.com/personal/lina4229_ox_ac_uk/Documents/Systematic%20Review/11768fc6-d4f5-4f1e-92db-c32686b5fd2c)  When viewing different nutrition content on social media, participants acknowledged the perpetuation of beauty standards and the importance of relatability (including the incorporation of local food products vs. international products not available in New Zealand), gatekeeping in nutrition, authenticity, and credibility.  “I think an issue at the moment is that there’s too much emphasis on perfection when it comes to health.”—young woman 3;  “I just find understanding nutrition like really difficult, like I just don’t get it”—young woman 8;  “Health is not all one size fits all, and it’s really, really difficult to navigate life constantly comparing yourself and your own health to what other people eat in a day or how much other people exercise, or how other people view themselves in their bodies.”—young woman12. |
|  | Perceptions that healthy eating campaigns/ ads do not target young adults and recommendations | [<Files\\Lambert et al. (2019) In their own words: A qualitative study exploring influences on the food choices of university students>](https://unioxfordnexus-my.sharepoint.com/personal/lina4229_ox_ac_uk/Documents/Systematic%20Review/a7f0ee7d-ebb5-4b3c-abdb-c3268656ab6c)  Reference 1  *Promotion campaigns:*  I don't think they target us. [MG3]  Reference 2  *Promotion campaigns:*  They use middle age people in those commercials…it doesn't feel relevant to us. [FG1]  Reference 3  *Regarding how best to deliver nutrition Information there was consensus it should be online incorporating social media:*  People our age don't watch TV…it's got to be online. [MG3]  Reference 4  *Regarding how best to deliver nutrition Information there was consensus it should be online incorporating social media:*  Social media because that's what we're exposed to the most – but the dietary guidelines aren't on social media. [FG3]  [<Files\\Molenaar et al. (2021) Effects of Advertising - A Qualitative Analysis of Young Adults' Engagement with Social Media About Food>](https://unioxfordnexus-my.sharepoint.com/personal/lina4229_ox_ac_uk/Documents/Systematic%20Review/6f6e9599-edb4-4423-b9db-c32686965524)  Reference 1  “I have recently seen many ads on [fast food brand name removed] mainly on Facebook, I ﬁnd it very annoying I also see it a lot on tv I never really see healthy food ads on tv or in any media I use daily.” (Forum 3: Female, 22 years old)  [<Files\\Molenaar et al. (2020) Language of Health of Young Australian Adults: A Qualitative Exploration of Perceptions of Health,](https://unioxfordnexus-my.sharepoint.com/personal/lina4229_ox_ac_uk/Documents/Systematic%20Review/a7f0ee7d-ebb5-4b3c-abdb-c3268656ab6c)  [Wellbeing and Health Promotion via Online Conversations>](https://unioxfordnexus-my.sharepoint.com/personal/lina4229_ox_ac_uk/Documents/Systematic%20Review/a7f0ee7d-ebb5-4b3c-abdb-c3268656ab6c)  Reference 1  Healthy eating and current healthy eating messages were sometimes perceived as boring and in need of a revamp and an element of fun to increase their appeal. Humour and the use of memes were seen as appealing attention-grabbing techniques that could be utilised. The involvement of peers was commonly mentioned as another fun campaign idea which would get people involved and invested due to the desire to fit in and be with their peers. Ideas included online challenges, and sharing fruit and vegetable consumption achievements through SM and community events.  To gain exposure from a large audience, it was seen as beneficial to create a “viral” campaign  related to fruit and vegetables on SM, with some mentioning the use of a catchy hashtag to help spread content. Going viral was important as it catches people’s attention, creates conversations and a new norm or culture for YA to engage in. Celebrities, SM influencers and athletes were suggested as potential spokespeople of the campaign.  “grocers should turn to sponsoring social media icons (government could do this too), particularly famous/popular instagram and youtube online celebrities who have vast followings. These should ideally be individuals who provide content that is health/food/exercise/sport/beauty centered, as the target audience would be those who have already recognised they want to lead a healthier lifestyle, who are in the process of change (or at least recognising that they need change).” (Challenge 1: Male, 22 years old)  [<Files\\Malloy et al. (2024) Empowering Young Women: A Qualitative Co-Design Study of a Social Media Health Promotion Programme. PDF>](https://unioxfordnexus-my.sharepoint.com/personal/lina4229_ox_ac_uk/Documents/Systematic%20Review/11768fc6-d4f5-4f1e-92db-c32686b5fd2c)  Young women in this study suggested that social media could be used to share “visual guides” for simple changes and healthy habits using exemplar local food products, underlining messages with a focus on addition rather than restriction (sustainable changes). Other suggestions included sharing easy ways to get in more exercise each day (for example, taking the stairs), simple and affordable recipes (including prices, ingredient lists, and instructions on the post), sharing information on the seasonality of foods, sharing other credible information sources to follow on social media and elsewhere on the internet, addressing mixed messages in nutrition, acknowledging the pitfalls of social media, direct messaging for accountability, authentic and relatable information (via infographics), acknowledgement of budgets and financial constraints, and inspiring reels. |
| SUSPICION TOWARDS FOOD PROMOTION STRATEGIES AND DIET ADVICE | Unconvinced by food promotion strategies | [<Files\\Molenaar et al. (2021) Effects of Advertising - A Qualitative Analysis of Young Adults' Engagement with Social Media About Food>](https://unioxfordnexus-my.sharepoint.com/personal/lina4229_ox_ac_uk/Documents/Systematic%20Review/6f6e9599-edb4-4423-b9db-c32686965524)  Reference 1  Some believed they could “tune out” any product promotions and therefore believed they never really saw advertisements. However, these participants who stated they did not really see advertisements were still able to remember and recall different advertisements when prompted in the forums.  “Hey Y’all, I also saw the new [Brand name removed] chocolate block—it really grabbed my attention. I think using bright colours will always get people’s attention and I don’t even fancy chocolate that much. I think of myself as ad- impenetrable. I often won’t buy products speciﬁcally because they are being advertised” (Forum 3: Male, 22 years old)  Reference 2  High frequency promotion was viewed as “annoying”, sometimes even considered “aggressive”, and generally decreased the likelihood that they would go and purchase the advertised food. “It played sometimes 3 times an ad break. Once I saw it back-to-back twice. It was exhausting.” (Forum 3: Female, 24 years old)  [<Files\\Buchanan et al. (2017) Exposure to digital marketing enhances young adults' interest in energy drinks - An exploratory investigation>](https://unioxfordnexus-my.sharepoint.com/personal/lina4229_ox_ac_uk/Documents/Systematic%20Review/e3cdf17c-185d-4bf8-92db-c32685e698da)  Reference 1  Only a minority of the participants took a more critical approach towards the nutritional claims made by the brands,  “I looked at the ingredients of both brands, so superficial, no scientific proof benefits. . . some of them are just a sentence, don’t believe that it will help me to concentrate at all”. (participant c, male, 22 years)  [<Files\\Vaterlaus et al. (2015) Getting Healthy - The percieved influence of social media on young adult health behaviours>](https://unioxfordnexus-my.sharepoint.com/personal/lina4229_ox_ac_uk/Documents/Systematic%20Review/aab09e5d-1410-45cb-91db-c32686d6fea2)  Reference 1  Finally, showing restraint after viewing a food post of chocolate cake, a participant (female, 21) said, ‘‘That looks good, but it’s not like I’m going to go buy a chocolate cake because of that.’’ |
|  | Distrust of diet-related advice/information | [<Files\\Brennan (2020) A social marketing perspective of young adults concepts of eating for health - Is it a question of morality>](https://unioxfordnexus-my.sharepoint.com/personal/lina4229_ox_ac_uk/Documents/Systematic%20Review/b2d0f086-d812-46f3-84db-c32685a6f2e7)  Reference 1  Others felt government-endorsed healthy eating messages were either false (“government is lying to me”) or over-exaggerated. In response to being shown a copy of the Australian Government’s Guide to Healthy Eating:  “When I look at this image, based off what I see before, I just think, "urgh", in resentment, as I have seen this so many times and I think the standards set out are just outrageous for a 'healthy lifestyle'. It's so hard to incorporate these foods into your daily life - and it's not a necessity, like, you're not going to be an obese person with heart disease if you don't follow the rules of the diet.” 24yo M  Reference 2  In response to a video advertisement about how fast food can contribute to central adiposity (increased fat around the waist):  “It slightly gross and also an exaggeration on how bad junk food is. It is bad but not that bad on moderation and if you exercise. I like the positive message associated with it that it is promoting unhealthy people to stop eating junk food. The negative is that it feels like a bit of an over exaggeration. I don't think it is aimed at me because even though I eat junk food a lot, I cover it up by exercising a lot. I wouldn't share this with my friends because we would end up laughing at this ad and eating junk food anyway.” 19yo M  [<Files\\Easton et al. (2018) - Young People's Experience of Viewing the Fitspiration SM Trend - Qualitative Study>](https://unioxfordnexus-my.sharepoint.com/personal/lina4229_ox_ac_uk/Documents/Systematic%20Review/bc617050-8526-44c3-8edb-c32686333bb0)  Reference 1  In fact, a few also believed that some of the diet-related material could even instigate an eating disorder, especially if they were unable to recognize that habits were becoming unhealthy.  If I followed their food account where they tell me to eat healthily and I couldn’t, I’d probably end up with an eating disorder. [P6, F, 20]  [<Files\\Leu et al.(2022)You know what, I'm in the trend as well-Understanding the interplay between digital & real-life social influences on the food & activity choices of YA>](https://unioxfordnexus-my.sharepoint.com/personal/lina4229_ox_ac_uk/Documents/Systematic%20Review/39ef073e-a1a8-49fd-94db-c32686754c65)  Reference 1  I mean, like, this pineapple tart, you can run for three days, it won’t go away. [participants laughed] And the thing is, they, they completely ignore, like, the calories you lose while you’re sleeping, that kind of thing :: : And they like to ::: .  Exaggerate ah. Fear monger.  – D01, male, 21, Chemical Engineering – D04, male, 24, Mechanical Engineering – D07, male, 24, Political Science  [<Files\\Lambert et al. (2019) In their own words: A qualitative study exploring influences on the food choices of university students>](https://unioxfordnexus-my.sharepoint.com/personal/lina4229_ox_ac_uk/Documents/Systematic%20Review/a7f0ee7d-ebb5-4b3c-abdb-c3268656ab6c)  Reference 1  Health promotion messages are so negative – like this is what happens to your gut when you drink coke. I know it's supposed to have an impact, but I don't feel like it has a significant impact. [FG3]  [<Files\\Molenaar et al. (2020) Language of Health of Young Australian Adults: A Qualitative Exploration of Perceptions of Health,](https://unioxfordnexus-my.sharepoint.com/personal/lina4229_ox_ac_uk/Documents/Systematic%20Review/a7f0ee7d-ebb5-4b3c-abdb-c3268656ab6c)  [Wellbeing and Health Promotion via Online Conversations>](https://unioxfordnexus-my.sharepoint.com/personal/lina4229_ox_ac_uk/Documents/Systematic%20Review/a7f0ee7d-ebb5-4b3c-abdb-c3268656ab6c)  Reference1  The media, including communications from the Government, and SM influencers, were seen to perpetuate misconceptions and conflicting messages of what is healthy which sometimes reduced YAs understanding of what a healthy diet looks like. Government messages such as the AGHE were often viewed as wrong or hard to believe, as it did not correlate with what they “knew” or other messages online. Some participants who followed an alternative diet to the AGHE, particularly those following a fad diet, did not trust the AGHE messages. These individuals had strong beliefs that their way of eating was what is “actually healthy”, specifically the exclusion of certain food groups—commonly carbohydrates and dairy. People sometimes viewed the guide as “one-size fits all” and not compatible with everyone and their different and specific needs—particularly those on a specific diet, e.g., plant-based, or those with certain allergies.  “I’d say it’s (the AGHE) an unrealistic representation of the “perfect diet” according to dietitians and doctors. Everybody’s diet is different, and everyone’s dietary requirement is different.” (Forum 14: Male, 19 years old)    Reference 2  SM was perceived as a constantly evolving culture of popular eating and exercise behaviours shaped by the rise of SM influencers and their idealised healthy lifestyles. These lifestyles portrayed on SM, although sometimes inconsistent with government health recommendations, were seen as ideal and something they should be following to fit in or be perceived favourably by peers. The comparison to the healthy ideal either motivated participants to change their behaviours to be more like the SM influencers or created a pressure to conform to these standards they often perceived as unachievable. Some YA reported feeling inadequate for not being able to reach these unattainable lifestyle behaviour goals.  “Too much of what is out there is coming from self-professed “gurus” and not from those with formal education. Alot of well being content is about looking “healthy” to impress people on Instagram, rather than what is actually good for you or realistic.” (Forum 4: Female, 24 years old).  [<Files\\Malloy et al. (2024) Empowering Young Women: A Qualitative Co-Design Study of a Social Media Health Promotion Programme. PDF>](https://unioxfordnexus-my.sharepoint.com/personal/lina4229_ox_ac_uk/Documents/Systematic%20Review/11768fc6-d4f5-4f1e-92db-c32686b5fd2c)  Reference 1  “I feel like with health. . . I find that there is a lot of different information, even if it’s with a credible source?”—young woman 13.  Reference 2  Some identified barriers to using social media included perpetuated beauty standards and harmful comparison, misleading or inaccurate nutrition and/or health messaging, and information overload.  “That is a real barrier—understanding what the standards are that are being pushed on us on social media vs. who am I?”—young adult 8;   “I feel like at some point it’s kind of like trying to confirm if I was correct? Because, you know, I might have some values, I might have some knowledge about this particular topic, but it’s just me and, you know, it will be really nice if I can look out to a platform that is so easily accessible, and there are people up there like, regardless of them being right or wrong or not, but you know we’re all on the same topic.”—young woman 2; |
|  | Budget-friendly fast food ads viewed as deceptive | [<Files\\Molenaar et al. (2021) Effects of Advertising - A Qualitative Analysis of Young Adults' Engagement with Social Media About Food>](https://unioxfordnexus-my.sharepoint.com/personal/lina4229_ox_ac_uk/Documents/Systematic%20Review/6f6e9599-edb4-4423-b9db-c32686965524)  Reference 1  “I dislike these [fast food] ads because they are misleading and don’t offer anything positive. Often working class people feel as though these foods are all they can afford, due to dollar menus and $5 meals, however it is consistently shown that whole foods are cheaper in the end.” (Forum 3: Female, 24 years old)  Reference 2  While value advertising strategies were appealing, and initially sounded like a cost- saving purchase, participants described how they usually end up buying more. This was especially mentioned in relation to fast-food companies with “meal deal”-type promotions. “The marketing they are using is to get people to visit the restaurants, people like myself are unlikely to spend only $3 because we consider this a good deal, it is likely that the more gullible of us (like myself) will spend more money than intended at the restaurant.” (Forum 3: Female, 24 years old)  Reference 3  Participants stated that advertisements that promoted deals were sometimes viewed as deceptive rather than the good value for money that was intended. Some participants were sceptical that special deals actually saved money and believed it was more likely to lure them in but then they would spend more than intended.  “Creating ‘meal deals’ is one of the best things fast food places have done. It makes people feel as though they are getting a good deal, while walking away having spent more money and eating more calories than they initially planned.” (Forum 3: Female, 24 years old) |

## **Table 12**: Risk of Bias Assessment – ROB1

| **STUDY ID** | **Study Design** | **Name of Cohort/**  **Study** | **Selection Bias – Random Sequence Generation** | **Selection Bias – Allocation Concealment** | **Performance Bias – Blinding of Participants & Outcome** | **Detection Bias – Blinding of Outcome Assessment** | **Attrition Bias – Incomplete Outcome Data** | **Reporting Bias – Selective Reporting** | **Other Bias** | **Overall Bias** |
| --- | --- | --- | --- | --- | --- | --- | --- | --- | --- | --- |
| Ahn (2015) | Randomised trial | Not stated | Low | Low | Unclear | Unclear | Low | Low | Low | Some concerns |
| Alblas (2018) | Randomised trial | Not stated | Unclear | Unclear | Unclear | Unclear | Low | Low | Unclear | Some concerns |
| Ashton (2017) | Randomised trial | ‘HEYMAN (Harnessing Ehealth to enhance Young men’s Mental health, Activity and Nutrition) | Low | Low | Low | Low | Low | Low | Low | Low |
| Buchanan (2017) | Randomised trial | Not stated | Low | Low | Low | Low | Low | Low | Low | Low |
| Duan (2017) | Randomised trial | Not stated | Unclear | Unclear | Unclear | Unclear | Low | Low | Low | Some concerns |
| Duan (2022) | Randomised trial | Not stated | Low | Low | Unclear | Unclear | High | Low | Low | High |
| Goodman (2016) | Randomised trial | Vitamin D Among Young Adults: an Intervention Study Using a Mobile 'App'. | Low | Low | Low | Unclear | Low | Low | High | High |
| Heikkilä (2019) | Randomised trial | Not stated | Unclear | Unclear | Low | Low | High | Low | Low | Some concerns |
| Kerr (2016) | Randomised trial | Connecting Health and Technology (CHAT) study | Low | Low | Unclear | Unclear | Low | Low | High | High |
| Kilb (2023) | Randomised trial | Not stated | Unclear | High | Unclear | Unclear | High | High | Low | High |
| Kinkel-Ram (2022) | Online experiment- randomised trial | Not stated | Low | Low | Low | Unclear | Low | Unclear | Low | Some concerns |
| Lai (2023) | Randomised trial | Not stated | Low | Low | Low | Low | Low | Low | Low | Low |
| Liu (2024) | Randomised trial | Not stated | Low | Low | Low | Low | Low | Low | Low | Low |
| Mackert (2012) | Randomised trial | Not stated | Unclear | Low | Low | Low | Low | Low | Unclear | Some concerns |
| Monroe (2015) | Randomised Trial | Green Eating Project | High | Unclear | Low | Low | Low | Low | Low | High |
| Mota (2023) | Randomised trial | My Viva Plan | Low | Low | Low | Low | Low | Low | Low | Low |
| O'Brien (2016) | Randomised Trial | N/A | Unclear | Unclear | Low | Low | Low | Low | Low | Some concerns |
| Park (2017) | Randomised Trial | Not stated | Unclear | Unclear | Low | Low | Unclear | Low | Low | Some concerns |
| Poddar (2010) | Randomised Trial | N/A | Unclear | Unclear | Unclear | Unclear | Low | High | Low | Some concerns |
| Plechatá (2022) | Randomised Trial | Long-term effects of IVR simulation on promoting sustainable eating behavior | Unclear | Low | Low | Low | Low | Unclear | Low | Some concerns |
| Priluck (2023) | Randomised trial | Not stated | Unclear | Unclear | Unclear | Unclear | Low | Low | Low | Some concerns |
| Schakel (2018) | Randomised Trial | Not stated | Low | Low | Unclear | Unclear | Low | Low |  | Some concerns |
| Schakel (2020) | Randomised Trial | Not stated | Low | Low | Unclear | Unclear | Low | Low |  | Some concerns |
| Whatnall (2019 | Randomised Trial | EATS - Eating Advice to Students | Low | Unclear | Low | Low | Unclear | Low | Low | Some concerns |

## **Table 13**: Risk of Bias Assessment – JBI Quasi-Experiment Checklist

| **STUDY ID** | **Study Design** | **Bias related to temporal precedence**  *Is it clear in the study what is the “cause” and what is the “effect” (i.e. there is no confusion about which variable comes first)?*  *Yes/No/Unclear/N /A* | **Bias related to selection and allocation**  *Was there a control group?*  *Yes/No/Unclear/ N/A* | **Bias related to confounding factors** *Were participants included in any comparisons similar?*  *Yes/No/Unclear/ N/A* | **Bias related to administration of intervention/exposure** *Were the participants included in any comparisons receiving similar treatment/care, other than the exposure or intervention of interest?*  *Yes/No/Unclear/ N/A* | **Bias related to assessment, detection and measurement of the outcome [list outcomes]**  *Were there multiple measurements of the outcome, both pre and post the intervention/exposure?*  *Yes/No/Unclear/ N/A* | **Bias related to assessment, detection and measurement of the outcome [list outcomes]**  *Were the outcomes of participants included in any comparisons measured in the same way?*  *Yes/No/Unclear/ N/A* | **Bias related to assessment, detection and measurement of the outcome [list outcomes]**  *Were outcomes measured in a reliable way?*  *Yes/No/Unclear/ N/A* | **Bias related to participant retention [list outcomes]**  *Was follow-up complete and if not, were differences between groups in terms of their follow-up adequately described and analyzed?*  *Yes/No/Unclear/ N/A* | **Statistical Conclusion Validity [list outcomes]**  *Was appropriate statistical analysis used?*  *Yes/No/Unclear/ N/A* | **Overall Appraisal** |
| --- | --- | --- | --- | --- | --- | --- | --- | --- | --- | --- | --- |
| Coccia (2020) | Intervention | Yes | No | Yes | Yes | Yes | Yes | Yes | Yes | Yes | Include |
| Chung (2021) | Controlled experimental study | Yes | Yes | Yes | Yes | Unclear | Yes | Yes | Yes | Yes | Include |
| Hawkins (2021) | Experiment | Yes | Yes | Yes | Yes | Yes | Yes | Yes | Yes | Yes | Include |
| Krishnamohan (2017) | Non-Randomised Trial | Yes | Yes | Yes | Yes | Yes | Yes | Yes | Yes | Yes | Include |
| Sharps (2019) | Non- Randomised Trial | Yes | No | Yes | Yes | Yes | Yes | Yes | Yes | Yes | Include |
| Slazus (2024) | Non- Randomised Trial | Yes | No | N/A | N/A | Yes | N/A | Yes | Yes | Yes | Include |

## **Table 14**: Risk of Bias Assessment – Newcastle Ottawa Scale

| **STUDY ID** | ***SELECTION—***  ***Representativeness of the sample:*** *a) Truly representative of the average in the target population. * (all subjects or random sampling) b) Somewhat representative of the average in the target population. * (non-random sampling) c) Selected group of users. d) No description of the sampling strategy.* | ***SELECTION—***  ***Sample size:*** *a. Justified and satisfactory (including sample size calculation). * b. Not justified. c. No information provided* | ***SELECTION—***  ***Non-respondents:*** *a) Comparability between respondents and non-respondents characteristics is established, and the response rate is satisfactory. * b) The response rate is unsatisfactory, or the comparability between respondents and non-respondents is unsatisfactory. c) No description of the response rate or the characteristics of the responders and the non-responders.* | ***SELECTION–***  ***Ascertainment of the exposure (risk factor):*** *a) Validated measurement tool. ** b) Non-validated measurement tool, but the tool is available or described.* c) No description of the measurement tool.* | ***COMPARABILITY –***  *1) The subjects in different outcome groups are comparable, based on the study design or analysis. Confounding factors are controlled. a) The study controls for the most important factor (select one). * b) The study control for any additional factor. ** | ***OUTCOME—***  ***Assessment of the outcome:*** *a) Independent blind assessment. ** b) Record linkage. ** c) Self report. * d) No description.* | ***OUTCOME—***  ***Statistical test:*** *a) The statistical test used to analyze the data is clearly described and appropriate, and the measurement of the association is presented, including confidence intervals and the probability level (p value). * b) The statistical test is not appropriate, not described or incomplete.* | ***TOTAL QUALITY SCORE—***  ***Risk of Bias Calculation****: High Quality Studies: 6-9 points Fair Quality Studies: 3-5 points Low Quality: 0-2 points* |
| --- | --- | --- | --- | --- | --- | --- | --- | --- |
| Buchanan (2018) | Low (one star) non-random sampling | High (no stars) No information provided | High (no stars) No information provided | Medium (one star) - Non validated tool but tool was described | Low (one star) - study controlled for important factor | Medium - one star. Self-report | Low - one star. Appropriate statistical test used | Fair quality |
| Hilkens (2021) | Low (one star) random sampling | High (no stars) not justified | High (no stars) - no information provided on non- respondents | Low (two stars) validated measurement | Low (one star) logistic regression analysis using randomisation was used to account for responses. | Medium - one star. Self-report | Low (one star) Appropriate statistical test used | High quality |
| Pollack (2022) | Low (one star) somewhat representative of the target population | High (no stars) not justified | Low - (two stars) response rate over 50% and comparability provided | Low (one star) - non-validated measurement tool but it is described | High - (No stars) the study did not control for anything | Low - validated tools utilised | Low - (one star) statistical test describes | High quality |

## **Table 15**: Risk of Bias Assessment – Critical Appraisal Skills Programme (CASP)

| **STUDY ID** | **STUDY DESIGN** | **1** | **2** | **3** | **4** | **5** | **6** | **7** | **8** | **9** | **10** | **OVERALL QUALITY** |
| --- | --- | --- | --- | --- | --- | --- | --- | --- | --- | --- | --- | --- |
| Brennan (2020) | Digital ethnography: web-based conversations | Yes | Yes | Yes | Yes | Yes | No | Yes | Yes | Yes | Yes | High |
| Buchanan (2017) | Semi-structured interviews (mixed methods) | Yes | Yes | Yes | Yes | Yes | Yes | Yes | Yes | Yes | Yes | High |
| Cardi (2012) | Case report and patient written feedback | Yes | No | Yes | Yes | Yes | No | No | Can't tell | Yes | Yes | Low |
| Duan (2022) | Interviews (mixed methods) | Yes | Yes | Yes | Yes | Yes | Can't tell | Yes | Can't tell | Can't tell | Yes | Medium |
| Easton (2018) | Focus groups and interviews | Yes | Yes | Yes | Yes | Yes | Can't tell | Yes | Yes | Yes | Yes | High |
| Friedman (2022) | Digital ethnography: web-based conversations | Yes | Yes | Yes | Yes | Yes | Yes | Yes | Yes | Yes | Yes | High |
| Lambert (2019) | Focus groups | Yes | Yes | Yes | Yes | Yes | No | Yes | Yes | Yes | Yes | High |
| Leu (2022) | Focus groups | Yes | Yes | Yes | Yes | Yes | No | Yes | Yes | Yes | Yes | High |
| Malloy (2024) | End-user co-design workshops | Yes | Yes | Yes | Yes | Yes | Yes | Yes | Yes | Yes | Yes | High |
| Molenaar (2020) | Digital ethnography: web-based conversations | Yes | Yes | Yes | Yes | Yes | Yes | Yes | Yes | Yes | Yes | High |
| Molenaar (2021) | Digital ethnography: web-based conversations | Yes | Yes | Yes | Yes | Yes | Yes | Yes | Yes | Yes | Yes | High |
| Nour (2019) | Semi-structured interviews (mixed methods) | Yes | Yes | Yes | Yes | Can't tell | No | Yes | Can't tell | Can't tell | Yes | Medium |
| Vaterlaus (2015) | Focus groups and interviews | Yes | Yes | Yes | Yes | Yes | Can't tell | Can't tell | Yes | Yes | Yes | Medium |
| Vedovato (2021) | Online questionnaires | Yes | Yes | Yes | Yes | Yes | No | Yes | No | Yes | Yes | Low |

## **Table 16**: GRADE-CERQUal summarised review of descriptive findings

| **#** | **Summarised review finding** | **GRADE-CERQual Assessment of confidence** | **Explanation of GRADE-CERQual Assessment** | **References of studies contributing to review finding** |
| --- | --- | --- | --- | --- |
| **BEHAVIOURAL RESPONSES** | | | | |
| 1 | Digital food communication enabled and supported participants to implement tactics and tools to eat healthy | High confidence | No/Very minor concerns regarding methodological limitations, No/Very minor concerns regarding coherence, No/Very minor concerns regarding adequacy, and No/Very minor concerns regarding relevance | Brennan et al., (2020); Molenaar et al., (2021); Nour et al., (2019); Vedovato et al., (2021); Duan et al., (2022) |
| 2 | Food purchases and dietary behaviours influenced by digital food communication in the form of advertisements | High confidence | No/Very minor concerns regarding methodological limitations, No/Very minor concerns regarding coherence, No/Very minor concerns regarding adequacy, and No/Very minor concerns regarding relevance | Molenaar et al., (2021); Friedman et al., (2022); Leu et al., (2022); Vaterlaus et al., (2015) |
| 3 | Peers influence dietary behaviours | High confidence | No/Very minor concerns regarding methodological limitations, No/Very minor concerns regarding coherence, No/Very minor concerns regarding adequacy, and No/Very minor concerns regarding relevance | Friedman et al., (2022); Leu et al., (2022); Vaterlaus et al., (2015); |
| **COGNITIVE RESPONSES** | | | | |
| 4 | Food choices are influenced by cost | High confidence | No/Very minor concerns regarding methodological limitations, No/Very minor concerns regarding coherence, No/Very minor concerns regarding adequacy, and No/Very minor concerns regarding relevance | Easton et al., (2018); Molenaar et al., (2021); Friedman et al., (2022) |
| 5 | Digital food communication presenting healthy eating information impacts awareness and motivation | High confidence | No/Very minor concerns regarding methodological limitations, No/Very minor concerns regarding coherence, No/Very minor concerns regarding adequacy, and No/Very minor concerns regarding relevance | Easton et al., (2018); Nour et al., (2019); Vedovato et al., (2021); Duan et al., (2022); Friedman et al., (2022); Leu et al., (2022); Vaterlaus et al., (2015) |
| 6 | Digital food communication in the form of interventions positively impact healthy eating considerations | Moderate confidence | Minor concerns regarding methodological limitations, No/Very minor concerns regarding coherence, Minor concerns regarding adequacy, and No/Very minor concerns regarding relevance | Cardi (2012); Duan et al., (2022); |
| 7 | Peers influence dietary considerations | High confidence | No/Very minor concerns regarding methodological limitations, No/Very minor concerns regarding coherence, No/Very minor concerns regarding adequacy, and No/Very minor concerns regarding relevance | Brennan et al., (2020); Friedman et al., (2022); Leu et al., (2022); |
| 8 | Young adults have differing perceptions of digital food communication in the form of food-related marketing and messages | High confidence | No/Very minor concerns regarding methodological limitations, No/Very minor concerns regarding coherence, No/Very minor concerns regarding adequacy, and No/Very minor concerns regarding relevance | Buchanan et al., (2017); Molenaar et al., (2021); Lambert et al., (2019); Friedman et al., (2022); |
| 9 | Young adults are suspicious of food promotion strategies and diet advice | High confidence | No/Very minor concerns regarding methodological limitations, No/Very minor concerns regarding coherence, No/Very minor concerns regarding adequacy, and No/Very minor concerns regarding relevance | Brennan et al., (2020); Buchanan et al., (2017); Easton et al., (2018); Molenaar et al., (2021); Lambert et al., (2019); Leu et al., (2022); Vaterlaus et al., (2015) |

Reporting Biases

*Behavioural Outcomes*

The funnel plot (Figure 5) indicates symmetry. The Egger’s test (p = 0.88) showed no evidence of small-study effects or publication bias in the funnel plot. The trim-and-fill analysis also supported the reliability of the meta-analysis findings.

## **Fig. 1**: Assessment of publication bias of studies focused on behavioural responses in meta-analysis

1. Positive significant findings are used to refer to significant findings for intended interventions [↑](#endnote-ref-2)
